# *In vivo* imaging of mammary epithelial cell dynamics in response to lineage-biased Wnt/β-catenin activation

**DOI:** 10.1016/j.celrep.2022.110461

**Published:** 2022-03-08

**Authors:** Bethan Lloyd-Lewis, Francesca Gobbo, Meghan Perkins, Guillaume Jacquemin, Mathilde Huyghe, Marisa M Faraldo, Silvia Fre

**Affiliations:** 1 Institut Curie, Laboratory of Genetics and Developmental Biology, PSL Research University, INSERM U934, CNRS UMR3215, F-75248 Paris Cedex 05, France; 2 School of Cellular and Molecular Medicine, University of Bristol, Biomedical Sciences Building, Bristol, BS8 1TD, UK

**Keywords:** mammary gland development, lineage tracing, β-catenin, Wnt Signaling, intravital imaging, *in vivo* imaging

## Abstract

Real-time, *in vivo* imaging provides an essential window into the spatiotemporal cellular events contributing to tissue development and pathology. By coupling longitudinal intravital imaging with genetic lineage tracing, here we capture the earliest cellular events arising in response to active Wnt/β-catenin signaling, and the ensuing impact on the organization and differentiation of the mammary epithelium. This enables us to interrogate how Wnt/β-catenin regulates the dynamics of distinct subpopulations of mammary epithelial cells *in vivo* and in real time. We show that β-catenin stabilization, when targeted to either the mammary luminal or basal epithelial lineage, leads to cellular rearrangements that precipitate the formation of hyperplastic lesions that undergo squamous transdifferentiation. These results enhance our understanding of the earliest stages of hyperplastic lesion formation *in vivo*, and reveal that in mammary neoplastic development, β-catenin activation dictates a hair-follicle/epidermal differentiation program independently of the targeted cell of origin.

## Introduction

The Wnt/β-catenin pathway is a fundamental and highly conserved signaling cascade that regulates tissue morphogenesis and stem cell fate in several tissues. Wnt signal activation results in the accumulation and nuclear translocation of β-catenin, which acts as a co-transcriptional activator of TCF/LEF target genes important for cell proliferation, survival and differentiation (MacDonald et al., 2009; Nusse and Clevers, 2017). In the absence of a Wnt ligand, the pathway remains inactive, with cytoplasmic levels of β-catenin maintained low by continuous proteasomal degradation (Nusse and Clevers, 2017; Steinhart and Angers, 2018).

Wnt/β-catenin signaling plays a central role at all stages of mammary gland development (Incassati et al., 2010; Jardé and Dale, 2012; Yu et al., 2016), and aberrant pathway activity is also widely implicated in breast tumorigenesis. Wnt-1, the prototype member of the Wnt family, was originally identified as a site of integration by the mouse mammary tumor virus (MMTV) (Nusse and Varmus, 1982), providing a link between Wnt signaling and breast cancer. Since, numerous studies have shown that dysregulated Wnt/β-catenin signaling leads to perturbed mammary gland development and tumorigenesis (reviewed in (van Schie and van Amerongen, 2020; Yu et al., 2016)). Targeted expression of Wnt-1 to the mammary luminal epithelium under the control of the MMTV long terminal repeats (LTR) results in a hyperbranched mammary phenotype and mammary adenocarcinomas (Li et al., 2003; Liu et al., 2004; Teissedre et al., 2009; Tsukamoto et al., 1988). Similarly, forced activation of Wnt signaling via MMTV-mediated expression of stabilized forms of β-catenin lacking N-terminal phosphorylation sequences (ΔN89- or ΔN90-β-catenin) results in precocious alveologenesis, and eventually adenocarcinomas (Imbert et al., 2001; Michaelson and Leder, 2001; Teissedre et al., 2009). Also, truncated β-catenin (ΔN57) targeted to the mammary basal compartment using the keratin K5 promoter induced basal-type hyperplasia in nulliparous aged females, in addition to squamous and invasive carcinomas in multiparous mice (Moumen et al., 2013; Teuliere et al., 2005).

In an alternative model, β-catenin stabilization can be achieved by Cre-mediated excision of loxP-flanked exon 3 of the endogenous *Catnb* gene *(Catnb^+/lox(ex3)^* mice) (Harada et al., 1999). Unlike models triggering exogenous β-catenin activation, *Catnb^+/lox(ex3)^* mice fail to develop mammary adenocarcinomas when β-catenin stabilization is induced in the luminal epithelium using whey acidic protein (WAP)-Cre (Pittius et al., 1988), or in all mammary epithelial cells with MMTV-Cre (Miyoshi et al., 2002b, 2002a). The impact of targeting mutant *Catnb^+/lox(ex3)^* specifically to the mammary basal epithelial compartment, and how this compares to phenotypes observed in luminal cells (Miyoshi et al., 2002b, 2002a), has yet to be investigated. As the molecular signatures and histopathological features of cancer cells do not necessarily reflect their presumptive cells of origin (Lim et al., 2009; Molyneux et al., 2010), studies focused on accurately dissecting the impact of the same oncogenic mutation in different cell types are warranted. Moreover, the early impact of sustained Wnt signaling on dynamic mammary epithelial cell behaviors and their neighboring wild-type cells, and how this affects the organization of the mammary gland during neoplastic transformation, is also largely unknown. Indeed, the rare nature of mutagenic events significantly hampers the *in situ* visualization of the earliest stages of cellular transformation. Thus, revealing the dynamic cellular mechanisms underlying this process promises to provide important insights into the critical steps leading to breast cancer initiation. To this end, we coupled genetic lineage tracing with longitudinal high-resolution intravital microscopy (IVM) to visualize the earliest changes in mammary luminal or basal epithelial cell dynamics in response to constitutive Wnt/β-catenin activation.

## Results

### Targeted stabilization of β-catenin to luminal mammary cells leads to hyperplastic lesions

**Fig. 1:**
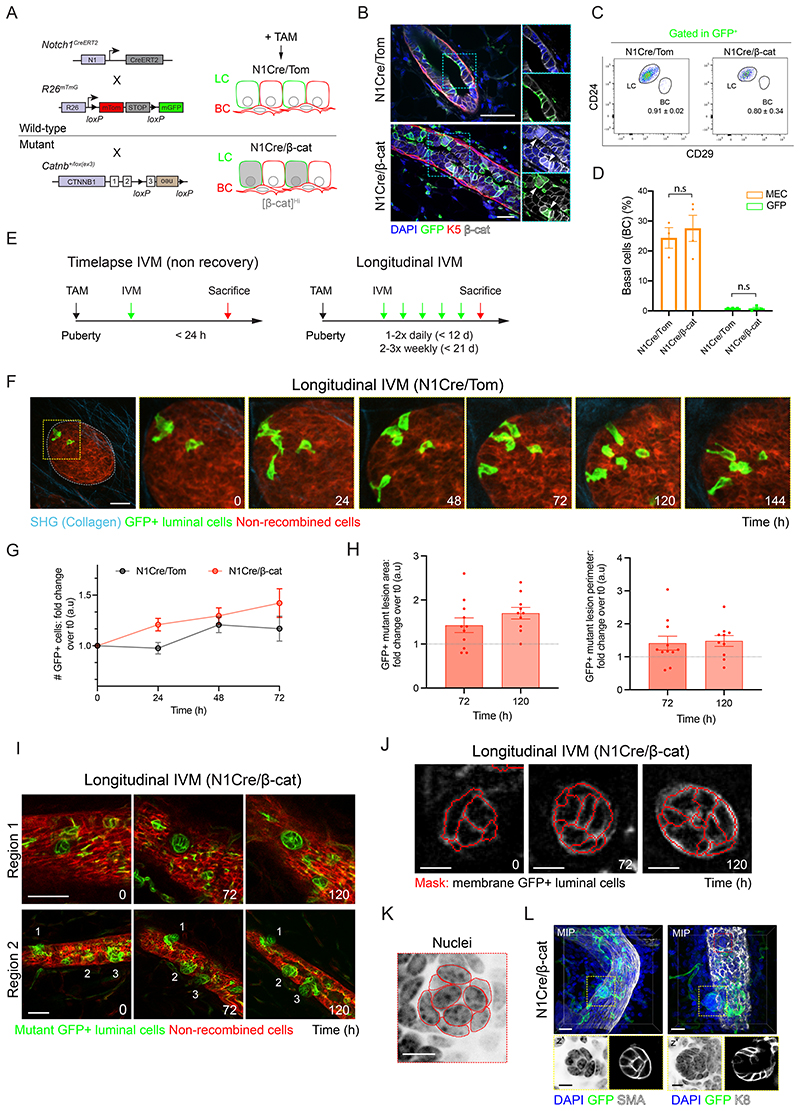
β-catenin activation in Notch1-expressing luminal cells induces mammary hyperplasia. **(A)** Schematic representation of the N1Cre/Tom and N1Cre/β-cat mice used in this study. All cells express membrane tdTomato fluorescence (red). Tamoxifen (TAM) administration induces membrane GFP (green) labelling of Notch1-expressing LC and their progeny and mutant β-catenin accumulation in N1Cre/β-cat mice (grey). **(B)** Representative sections of mammary glands showing β-catenin cytoplasmic/nuclear accumulation coinciding with membrane GFP expression (arrowheads) in N1Cre/β-cat luminal cells 7 days after Cre induction. K5 is shown in red, anti-β-catenin staining in white and DAPI labels nuclei in blue. Scale bars: 20μm. **(C)** Representative FACS dot plots of luminal CD24^+^CD29^lo^ (LC) and basal CD24^+^CD29^hi^ (BC) mammary cells gated within GFP+ mammary epithelial cells (MEC) in pubertal N1Cre/Tom and N1Cre/β-cat mice 48-72h after Cre induction. GFP+ MECs are restricted to the luminal compartment in both models. Average values are shown ± SEM. **(D)** Quantification of GFP+ BC (green) as compared to the proportion of total BCs within MECs (orange) in N1Cre/Tom or N1Cre/β-cat mice 48-72 h after Cre induction. Graph shows mean ± SEM. No statistical differences (n.s) were observed between transgenic lines (p > 0.05, Welch’s t-test, n=3-4 mice/group). **(E)** Schematic representation of experimental timelines for time-lapse or longitudinal intravital imaging in wild-type and β-catenin mice. **(F)** IVM images of a mammary terminal end bud (TEB) in a pubertal N1Cre/Tom mouse showing recombined GFP+ (green) LC rearrangements over 6 days (144 h). Red: non-recombined tdTomato+ cells, cyan: collagen (SHG). t0 refers to 48 h after TAM administration. Related to Supplemental Movie 1. Scale bar: 50μm. **(G)** Quantification of number of GFP+ cells in TEBs over time. 9 structures in 4-5 mice per genotype were imaged over 72 h by longitudinal IVM. (p = 0.124 between groups, two-way RM ANOVA). **(H)** Graphs depicting the increasing size of mutant GFP+ lesions over time, plotted as fold increase over t0. n=11 lesions analyzed in 3 mice (p = 0.0015 (area) and 0.0556 (perimeter), mixed effects model). **(I)** IVM images of a mammary duct in a N1Cre/β-cat mouse showing the development of hyperplastic luminal GFP+ (green) lesions over time, n=3 mice. t0 represents 16 days after Cre induction. Red: non-recombined tdTomato+ cells. Scale bar: 50μm. **(J)** Segmentation masks of mutant GFP+ cells in a developing lesion (Fig 1I, Region 1) imaged over time by IVM. Scale bar: 10μm. **(K)** Segmentation masks of DAPI-stained nuclei displayed in L (right panel, red box). Scale bar: 10 μm. **(L)** Maximum intensity z- projection (MIP) of cleared mammary tissues from N1Cre/β-cat mice marked with anti-SMA (left) or anti-K8 (right) in white, 2-3 weeks after Cre induction. Insets (z) show DAPI staining (left) and GFP+ fluorescence (right) in a single optical slice. Scale bar: 20μm (MIP), 10μm (insets).

The mammary ductal network is composed of two main epithelial lineages: basal (BC) and luminal (LC) cells, with the latter subdivided based on the expression or absence of the hormone receptors estrogen receptor-α (ERα) and Progesterone Receptor (PR). Notch signaling is a critical determinant of luminal cell fate (Bouras et al., 2008; Lilja et al., 2018), with Notch1 receptor expression restricted to ERα/PR-negative luminal progenitor cells in the postnatal mammary gland (Rodilla et al., 2015). Thus, to investigate the impact of constitutive Wnt/β-catenin activation on LC, we crossed *Catnb^+/lox(ex3)^* mice (Harada et al., 1999) to Notch(N)1Cre^ERT2^;R26^mTmG^ mice (Rodilla et al., 2015) (Fig.1A, henceforth referred to as N1Cre/Tom). In these compound mice, the N1-Cre^ERT2^ line (Fre et al., 2011) is crossed to the double fluorescent reporter model Rosa26^mTmG^ (Muzumdar et al., 2007), enabling membrane-bound tdTomato expression to be switched to membrane-bound green fluorescent protein (GFP) in Notch1-expressing cells and their progeny upon Tamoxifen (TAM) administration (Fig. 1B, S1A) (Rodilla et al., 2015). While β-catenin was predominantly restricted to epithelial basolateral membranes in wild-type mice, Cre induction in mutant N1-Cre^ERT2^;R26^mTmG^;*Catnb^+/lox(ex3)^* mice (henceforth referred to as N1Cre/β-cat) resulted in β-catenin accumulation in mammary LC that correlated with GFP expression, thus representing a robust indicator of mutant β-catenin status (Fig. 1B). As expected (Lilja et al., 2018; Rodilla et al., 2015), flow-cytometry analyses confirmed the localization of Notch1-derived GFP+ mammary epithelial cells to the luminal compartment (CD24^+^CD29^low^) (Fig.1C, D Fig.S1B).

To visualize cellular dynamics *in vivo* and *in situ* during mammary ductal development, we surgically implanted an imaging window (Jacquemin et al., 2021; Zomer et al., 2013) over the abdominal (4^th^) mammary gland of 5/6-week-old pubertal mice, 24-72 h after Cre induction (Fig. 1E). This approach enabled the high-resolution 4-dimensional (x-, y-, z- t-) intravital imaging of the mammary epithelium over time in physiological conditions. Consistent with recent short-term IVM studies using neutral labelling strategies (Corominas-Murtra et al., 2020; Messal et al., 2021; Scheele et al., 2017), longitudinal IVM in pubertal mammary glands of N1Cre/Tom mice revealed the cellular rearrangements of terminal end bud (TEB)-resident LC (Fig. 1F, S1C, Movie S1), validating our *in vivo* imaging conditions. Interestingly, Notch1-derived GFP+ LC in stratified TEBs intermittently, but regularly, extended cellular protrusions to contact the basal epithelial layer over time, exposing them to signals from the basement membrane (Fig. 1F, S1C, Movie S1), as previously observed in fixed tissues (Lafkas et al., 2013) and by time-lapse imaging of mammary organoids (Ewald et al., 2012). Notably, these cells were maintained at the distal tips of TEBs, and not deposited in subtending ducts during branching morphogenesis. Notch1-derived GFP+ LC in ductal structures were also observed to dynamically interact with the basal compartment by longitudinal IVM spanning several days (Fig. S1D). This serial imaging approach also revealed at an unprecedented resolution the dynamic process of mammary lumen formation *in vivo*, whereby fusion of several discrete lumina drives the establishment of a ductal network (Fig.S1E-F).

Our ability to visualize the cellular dynamics of individual mammary cells *in situ* over time by IVM provided a powerful platform with which to interrogate the early impact of sustained Wnt signaling on the dynamics of specific luminal or basal mammary cells. We therefore coupled the lineage tracing of β-catenin gain-of-function mice with intravital imaging of mammary epithelial cell dynamics. At early time-points after β-catenin activation (up to 72h), we did not observe significant differences between wild-type and mutant clonal expansion by longitudinal IVM (Fig. 1G), and the mammary epithelium appeared morphologically normal at this stage (Fig 1B, S2A, Movies S2-4).

Next, to visualize the long-term impact of Wnt/β-catenin activation on LC dynamics, we performed longitudinal IVM in the mammary glands of pubertal N1Cre/ β-cat mice for up to 3 weeks (Fig. 1E). This approach revealed the dynamic rearrangement of GFP+ mutant cells into compact circular lesions that expanded on average 1.7-fold over 5 days, rapidly encroaching neighboring basal cells (Fig. 1H-I) (t0 = 16 days after Cre induction). We observed GFP+ mutant cells clustering in a circular arrangement (Fig. 1I-J), and their divergent displacement towards newly forming bud-like structures (Fig. 1I Region 2, S2B). Downstream immunostaining and 3D imaging further confirmed the organization of epithelial nuclei into compact, circular structures (Fig. 1K) and their orientated arrangement in developing buds (Fig. 1L). These findings suggest that, similar to observations in adult mouse skin in response to β-catenin activation in hair follicle stem cells (Deschene et al., 2014), constitutive activation of β-catenin orientates and organizes cell movements in a manner that leads to the development of aberrant growths. Lesions with empty central regions were also observed, suggesting that internal cells within developing buds undergo cell death (Fig. S2C, 19-21 days post induction), which was supported by cleaved caspase-3 (CC3) immunostaining and terminal transferase-mediated dUTP nick end labelling (TUNEL) of early and advanced lesions in harvested mammary tissues (Fig.S2D-F). Collectively, these findings show that activated Wnt signaling promotes LC rearrangements to drive the development of hyperplastic lesions.

### Targeted stabilization of β-catenin to basal mammary cells also leads to abnormal epithelial budding and hyperplastic lesions

**Fig. 2:**
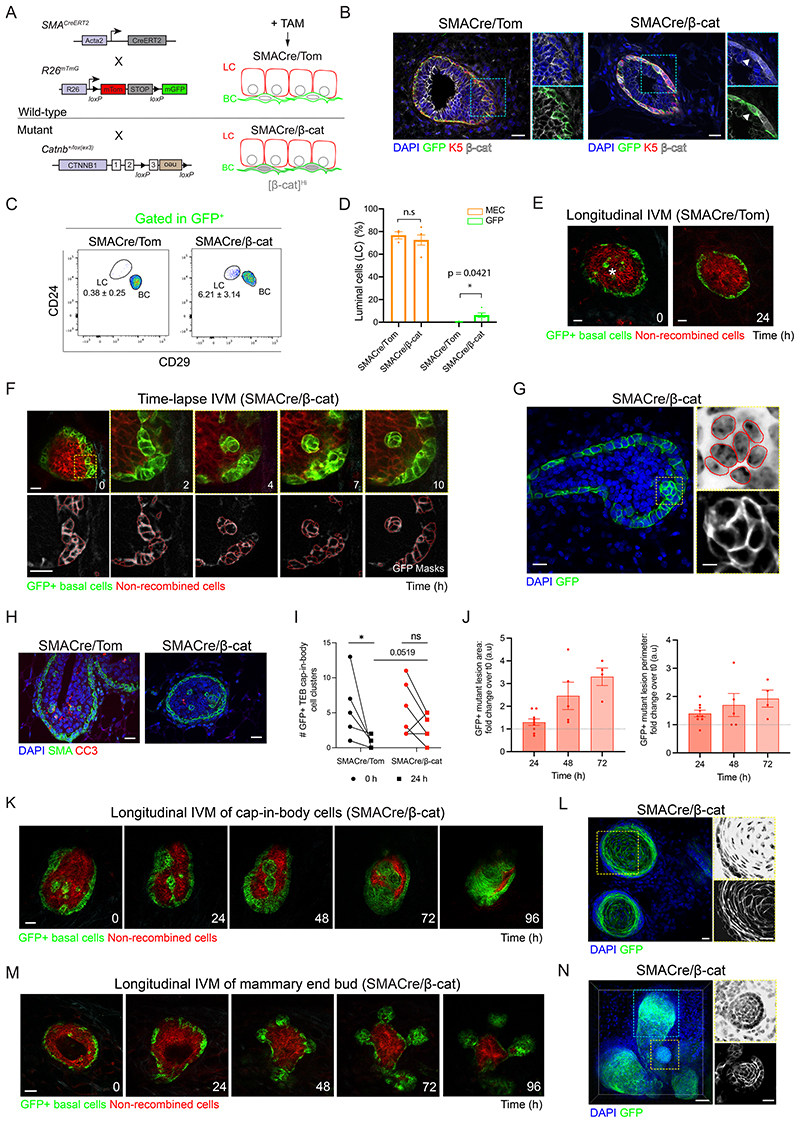
β-catenin activation in basal cells induces hyperplastic lesions. **(A)** Schematic representation of the SMACre/Tom and SMACre/β-cat mouse models used. All cells are labelled with a red membrane tomato fluorescence. Tamoxifen (TAM) administration induces membrane GFP labelling of basal cells and β-catenin accumulation in the SMACre/β-cat model. **(B)** Representative mammary sections showing β-catenin nuclear accumulation in K5-expressing basal cells in SMACre/β-cat mice, coinciding with membrane GFP expression (arrowheads). K5 is shown in red, anti-β-catenin staining in white and DAPI labels nuclei in blue. Scale bar: 20μm. **(C)** Representative FACS dot plots of luminal (LC) and basal (BC) mammary cells gated within GFP+ SMACre/Tom and SMACre/β-cat mammary epithelial cells 48-72h after Cre induction. Average values ± SEM are shown. **(D)** Quantification of GFP+ LC (green) as compared to the proportion of LCs within MECs (orange) in SMACre/Tom or SMACre/β-cat 48-72 h after Cre induction. Graph shows mean ± SEM (* p=0.0421, Welch’s t-test, n=3-5 mice per group). **(E)** IVM images of a terminal end bud (TEB) in a SMACre/Tom mouse showing the elimination of basal GFP+ (green) cap-in-body cells (asterisk) within 24 h. n=4 mice. Scale bar: 25μm. **(F)** Acute time-lapse IVM of basal GFP+ (green) cap-in-body cells in a TEB of a pubertal SMACre/β-cat mouse. n=2 mice. Bottom panel shows the segmentation masks of GFP+ cells. Scale bar: 20μm. **(G)** Optical section of a TEB 72 h after Cre induction showing DAPI staining in blue (nuclei outlined in red in the top inset) and GFP fluorescence in green (bottom inset). Scale bar: 20μm (main), 5 μm (inset). **(H)** Representative sections of mammary tissues from pubertal SMACre/Tom and SMACre/β-cat mice immunolabeled for anti-cleaved-caspase 3 (CC3, red) in TEBs. SMA expression (green) marks internalized cap-in-body basal cells. Scale bar: 20μm. **(I)** Graph showing the number of GFP+ cap-in-body cell clusters in distinct TEBs in pubertal SMACre/Tom and SMACre/β-cat mice at the start of imaging (t0) and 24 h later. 30-33 cell clusters were imaged at t0 in 6 different TEBs from 4-5 mice per group. (*p = 0.0312, ns: not significant; Wilcoxon paired t-test. p=0.0519 between genotypes at t 24 h; Mann Whitney test). **(J)** Graphs depicting the increasing size of mutant GFP+ lesions over time, plotted as fold increase over t0. n=9 discrete lesions in 4 different mice analyzed every 24 h for 3 days, or until clone convergence. p = 0.0025 (area) and 0.0261 (perimeter), mixed effects model. **(K)** IVM images showing the expansion of GFP+ (green) basal cap-in-body cells in a SMACre/β-cat TEB over time. Scale bar: 50 μm. **(L)** Confocal image of TEBs 2 weeks after Cre induction showing DAPI staining (top inset) and GFP fluorescence (bottom inset). Scale bar: 20μm. **(M)** IVM images showing aberrant bud formation over time in SMACre/β-cat pubertal mammary glands. n=5 mice. Scale bar: 50 μm. **(N)** Maximum intensity z-projection (MIP) of cleared mammary tissues from SMACre/β-cat mice 2 weeks after low-dose TAM. Yellow outlined insets show DAPI staining (top) and GFP+ fluorescence (bottom) in a single optical slice. Scale bar: 50μm (MIP), 20μm (insets). Area outlined by the blue box is shown in Fig. S3G.

To induce β-catenin stabilization specifically in BC, we crossed the same *Catnb^+/lox(ex3)^* transgenic line (Harada et al., 1999) to Acta2-Cre^ERT2^ (SMA-Cre^ERT2^) (Wendling et al., 2009);R26^mTmG^ mice (henceforth referred to as SMACre/Tom and SMACre/β-cat for wild-type and mutant lines, respectively) (Fig.2A). As expected, TAM administration in SMACre/Tom and SMACre/β-cat mice led to GFP expression in the basal cell layer (Fig. 2B, S3A-B). Flow-cytometry analyses 48-72 h after Cre induction confirmed the confinement of SMA-derived GFP+ mammary cells to the basal compartment (CD24^+^CD29^hi^) in SMACre/Tom control mice (Fig.2C-D). By contrast, a minor percentage of GFP+ cells isolated from SMACre/β-cat mammary glands were also detected in the luminal compartment (CD24^+^CD29^lo^), suggesting perturbation of normal mammary epithelial lineage segregation in response to constitutive β-catenin stabilization within 72 h (Fig.2C-D).

To visualize the impact of β-catenin stabilization on BC dynamics, we next performed short-term IVM in SMACre/Tom and SMACre/β-cat pubertal mammary glands 24-72 h after Cre induction. Using this approach, we documented the rapid removal of wild-type GFP+ BC (SMACre/Tom) residing in the body cell layer of TEBs (referred to as “cap-in-body” cells) (Fig. 2E, S3C). In contrast, we observed the dynamic re-arrangement of mutant β-catenin cap-in-body cells (Fig. 2F, Movie S5) analogous to those observed in the N1Cre/β-cat model (Fig.1J-K). DAPI staining of fixed tissues confirmed the re-arrangement of epithelial nuclei into compact, circular structures (Fig.2G). CC3 immunostaining also suggested decreased cell death in mutant compared to wild-type TEBs (Fig.2H, S3D). While no significant differences were observed in the size of wild-type and mutant GFP+ cap-in-body cell clusters at the start of imaging (Fig.S3E), quantitative analysis of their fate by longitudinal IVM revealed that approximately 83% of cap cells residing in wild-type TEBs were eliminated within 24 h (Fig.2I, S3F), consistent with recent time-lapse IVM studies (Dawson et al., 2021). By contrast, nearly 50% of mutant β-catenin GFP+ cap-in-body cells detected at the start of imaging were retained after 24h (Fig.2I, S3F). Sequential IVM over several days revealed that surviving mutant GFP+ cap-in-body BC rapidly expanded to form dysplastic squamous-like nodules (Fig. 2J-K, Movie S6) encompassing pleomorphic nuclei arranged in a ring-like arrangement (Fig.2L, S3G). Moreover, while wild-type GFP+ BC retained their typical elongated morphology in TEBs and ducts (Fig.S4), mutant GFP+ BC became increasingly cuboidal in shape (Fig. 2G, K). Interestingly, mutant BC swiftly gave rise to ectopic bud-like structures (Fig. 2M) that resembled the assemblies observed with luminal targeting of mutant β-catenin (Fig. 1I, S2B). Indeed, downstream nuclear staining showed a similar upward re-orientation of epithelial nuclei within developing growths (Fig. S3H). Cells within established ectopic buds were often organized in a circular arrangement (Fig. 2M-N insets), implying that these may eventually acquire the same dysplastic squamous-like phenotype rapidly arising in neighboring TEBs (Fig. 2N, S3G) at later time points. GFP fluorescence within lesions gradually diminished over time (Fig. 2K, M, Movie S6), with numerous apoptotic cells detected in both early and advanced lesions by TUNEL and CC3 immunostaining (Fig.S3I-J).

### Constitutive Wnt/β-catenin-induced lesion formation is mediated by hyperproliferation

**Fig. 3:**
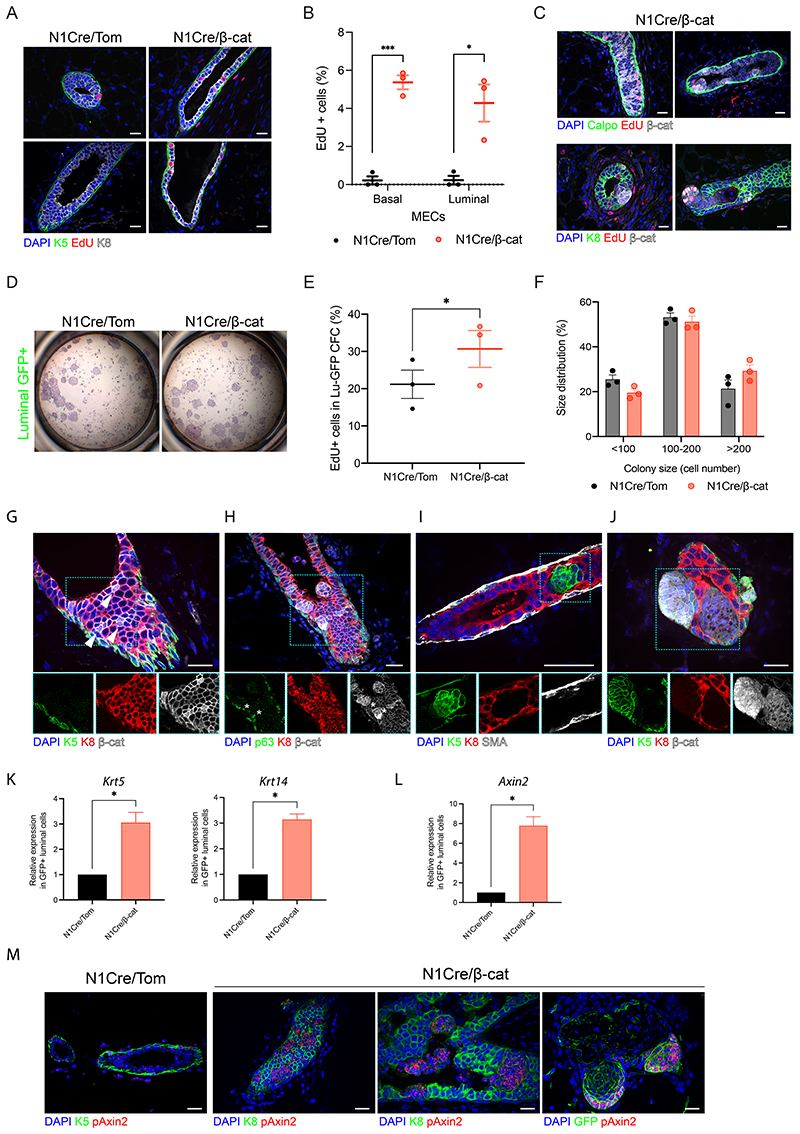
β-catenin stabilization in luminal cells leads to increased proliferation and aberrant lineage marker expression. **(A)** Representative sections of N1Cre/Tom and N1Cre/β-cat mammary glands showing EdU+ (red) cells in the luminal (K8, white) and basal (K5, green) compartment. **(B)** Quantification of the percentage of EdU+ (GFP+ or GFP-) cells in BC and LC in phenotypically normal WT and mutant β-catenin ducts. Graph shows mean ± SEM (* p < 0.05, *** p = <0.001 Welch’s t-test; n = 3 animals per group, 3 weeks after Cre induction). **(C)** Edu+ cells (red) in N1Cre/β-cat mammary sections coincided with β-catenin accumulation (white) in nascent and established lesions 6-7 days after Cre induction. **(D)** No differences were observed in the colony forming capacities of GFP+ LC isolated from N1Cre/Tom and N1Cre/β-cat mammary glands 3 weeks after Cre induction. Representative images of hematoxylin and eosin-stained colonies after 7 days in culture. **(E)** Colonies arising from GFP+ LC isolated from N1Cre/β-cat mice possessed a higher percentage of proliferative (EdU+) cells. Graph shows mean ± SEM (*p < 0.05, paired t-test; n=3 independent sorting experiments). **(F)** Size distribution of colonies generated from WT or mutant GFP+ LC. Graph shows mean ± SEM (p < 0.05, Pearson’s chi-square test; n=3 independent experiments performed 3 weeks after induction). **(G-J)** Representative sections of mammary ducts immunostained for β-catenin (white in G, H, J) in N1Cre/β-cat mice, showing changes in lineage marker expression. β-catenin accumulation (marked by white arrows) in luminal K8 expressing cells (red) in a phenotypically normal duct 6 days after Cre induction **(G)**. The asterisks in **(H)** show aberrant expression of basal marker p63 (green) in early luminal K8+ lesions 6-7 days after Cre induction. **(I-J)** Larger mutant β-catenin derived lesions are devoid of K8 (red) expression and express K5 (green), a mammary basal marker, 3 weeks after Cre induction. **(K, L)** RT-qPCR analysis of *Krt5, Krt14* **(K)** and *Axin2* **(L)** expression in sorted GFP+ luminal cells from N1Cre/Tom and N1Cre/β-cat females. Graphs show means ± SEM (p < 0.05 Welch’s t-test; n=3 independent experiments). **(M)** Representative sections of N1Cre/Tom (left panel) and N1Cre/β-cat mammary tissues analyzed by smRNA FISH (RNAscope) for *Axin2* (pAxin2 in red) and immunostained for K5 (N1Cre/Tom), K8 or GFP (N1Cre/β-cat) (green), as indicated. Scale bar: 20 μm.

Acute and longitudinal IVM imaging revealed the dynamic rearrangements and expansion of LC or BC in response to β-catenin stabilization, with similarities observed in the organization of mutant cells during the early stages of lesion development between both models. To investigate the cellular mechanisms underlying the observed phenotypes, we characterized the proliferative capacity of mutant β-catenin cells by 5-ethynyl-2'-deoxyuridine (EdU) incorporation. While rarely detected in N1Cre/Tom mammary epithelium (Fig. 3A), EdU+ cells were markedly increased in N1Cre/β-cat mammary glands, even within morphologically normal ducts (Fig. 3A-B). Surprisingly, increased EdU uptake was observed in both luminal and basal compartments prior to lesion development, suggesting a non-cell autonomous effect (Fig. 3B). The majority of EdU+ wild-type cells resided 1-2 cells from mutant β-catenin clones (Fig. S5A), indicating that paracrine signals are likely short-range. EdU incorporation correlated with β-catenin accumulation both in morphologically normal ducts and in nascent luminal lesions (Fig. 3C), further indicating that increased proliferation contributed to the cellular dynamics observed by IVM.

To investigate if β-catenin activation affected the clonogenic capacity of Notch1-expressing luminal progenitors, GFP+ LC from N1Cre/Tom and N1Cre/β-cat mice were isolated by flow cytometry and seeded on a feeder layer of irradiated 3T3 fibroblasts (Sleeman et al., 2007). No differences were observed between the colony-forming potential of wild-type and mutant LC, suggesting that progenitor cell frequencies were comparable (Fig. 3D, Fig.S5B). Mutant colonies contained a higher proportion of proliferating cells, however, making larger colonies and corroborating our *in vivo* observations (Fig. 3E-F).

We next sought to molecularly characterize the lesions arising in response to luminal targeting of β-catenin in N1Cre/β-cat mice. While restricted to basolateral membranes in wild-type mammary cells (Fig.S5C), β-catenin accumulation was clearly visible in the cytoplasm of K8-expressing LC in N1Cre/β-cat mammary glands shortly after Cre induction (Fig.3G). β-catenin stabilization was concentrated in nascent luminal lesions that retained luminal marker expression, although rare p63- and Id4-expressing LC (K8+) were also observed (Fig. 3H, S5D). Over time, Notch1-derived lesions frequently appeared to lose K8 staining and gain expression of the basal cytokeratins K5 and K14 (Fig. 3I-K), suggesting the acquisition of basal characteristics in response to Wnt activation. Increased proliferation in the luminal compartment appeared restricted to hormone receptor negative cells (Fig. S5E). Of interest, these lesions invariably lacked the expression of the BC marker SMA (Fig. 3I, S5D). Moreover, clusters of basally-located, K5-expressing cells with intense nuclear β-catenin staining were frequently observed next to inner cell clusters that exhibited weaker staining (Fig. 3J). RT-qPCR analysis confirmed the strongly increased expression of the Wnt target gene, *Axin2*, in mutant β-cat LC compared to wild-type cells (Fig. 3L). Single molecule RNA FISH (smRNA FISH) analysis with an Axin2 probe also showed the dynamic accumulation of *Axin2* transcripts, which was visible in LC even before lesion formation and substantially increased with time (Fig. 3M). Lesions eventually developed into large squamous metaplastic-like structures that encircled islands of keratin debris and cells with pleomorphic or absent nuclei (“ghost cells”) (Fig. S5F), consistent with previous observations of constitutive Wnt/β-catenin activation (Jardé et al., 2016; Miyoshi et al., 2002b, 2002a). Lesion development and transition to squamous metaplasia proceeded similarly when inducing mutant β–catenin activity in older N1Cre/β-cat mice (Fig. S5G).

**Fig. 4:**
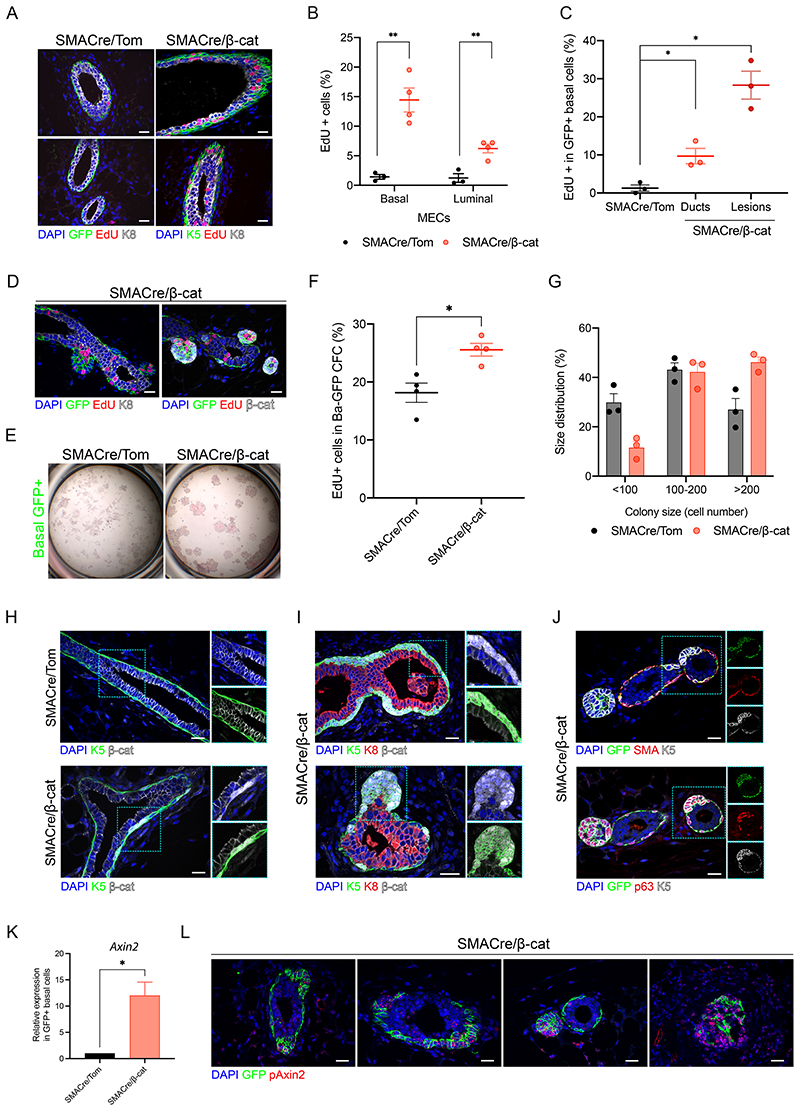
β-catenin stabilization in basal cells leads to increased proliferation and aberrant lineage marker expression. **(A)** Representative sections of SMACre/Tom and SMACre/β-cat mammary glands showing EdU+ (red) cells in the luminal (K8, white) and basal (K5 or GFP, green) compartments. **(B)** Quantification of the percentage of EdU+ cells in BC and LC in phenotypically normal SMACre/Tom and SMACre/β-cat ducts. Graph shows mean ± SEM (** p < 0.01 Welch’s t-test; n=3 animals per group). **(C)** Quantification of proliferative GFP+ BC in SMACre/Tom mammary sections, normal ducts and aberrant regions in SMACre/β-cat mice. Graph shows mean ± SEM (* p < 0.05, Welch’s t-test; n=3). **(D)** Edu+ cells (red) in mutant SMACre/β-cat mammary tissue sections coincided with GFP expression and β-catenin accumulation (white) in mammary lesions. **(E)** No statistically significant differences were observed in the colony forming capacities of GFP+ BC isolated from SMACre/Tom and SMACre/β-cat mammary glands. Representative images of hematoxylin and eosin-stained colonies after 7 days in culture. **(F)** Colonies arising from GFP+ BC isolated from SMACre/β-cat mice present a higher percentage of proliferative (EdU+) cells. Graph shows mean ± SEM (* p < 0.03, paired t-test; n=4 independent experiments). **(G)** Size distribution of colonies generated from WT and mutant GFP+ LC. Graph shows mean ± SEM. p <0.0001, Pearson’s chi-square test; n=3 independent experiments. **(H)** Representative sections of mammary ducts showing β-catenin (in white) cytoplasmic/nuclear accumulation in K5-expressing basal cells (in green) in the mammary epithelium of SMACre/β-cat mice. **(I)** Representative images of mammary sections showing β-catenin-induced changes to BC morphology and the development of aberrant bud-like lesions in SMACre/β-cat mice. β-catenin (in white), K5 (in green) and K8 (in red), as indicated. **(J)** Aberrant basal-derived lesions express K5 (in white) and p63 (in red, lower panel) but lack SMA expression (in red, upper panel). **(K)** RT-qPCR analysis of *A×in2* expression in sorted GFP+ BC from SMACre/Tom and SMACre/β-cat glands. Graph shows mean ± SEM (p < 0.02 Welch’s t-test; n=4 independent experiments). **(L)** Representative sections of SMACre/β-cat mammary tissues analyzed by smRNA FISH for *A×in2* (pAxin2 in red) and immunostained for GFP (green). Related to Fig.S6D. Scale bar: 20 μm.

Analogous EdU incorporation studies in SMACre/Tom and SMACre/β-cat mice also revealed a marked increase in the proportion of EdU+ BC prior to lesion development (Fig. 4A). Notably, like in N1Cre/β-cat mice, increased proliferation was also detectable in neighboring LC in morphologically normal ducts, indicative of short-range paracrine signaling (Fig. 4B, S6A). The high rate of recombination induced by *SMA-Cre^ERT2^* enabled us to quantify the proportion of proliferating GFP+ cells in wild-type and mutant mice, revealing a 10-fold increase in the mutant epithelium, rising to nearly 30-fold in visible lesions (Fig. 4C). EdU incorporation was particularly evident in cells with β-catenin accumulation in mutant-derived lesions (Fig. 4D). In line with our results in luminal targeted cells, β-catenin activation had no significant impact on the *in vitro* colony-forming efficiency of GFP+ BC (Fig.4E, S6B). Mutant colonies were more proliferative, however, and significantly larger than control colonies (Fig.4E-G).

While no cytoplasmic/nuclear β-catenin could be observed in BC of SMACre/Tom mice, SMACre/β-cat BC exhibited strong nuclear β-catenin staining and a more cuboidal morphology (Fig.4H-I). Although lesions retained the expression of the basal markers K5 and p63, they invariably lacked SMA expression, a marker for myoepithelial differentiation (Fig.4I-J). As observed by IVM (Fig. 2), mutant growths rapidly evolved into large dysplastic squamous-like structures containing ghost cells lacking mammary epithelial marker expression (Fig. S6C). Like in the N1Cre model, β-catenin stabilization resulted in Wnt pathway activation, as shown by the strong increase in *Axin2* expression by RT-qPCR (Fig. 4K). smRNA FISH also revealed the accumulation of *Axin2* transcripts alongside cellular proliferation and lesion growth (Fig. 4L, S6D). Lesions arising upon induction of mutant β-catenin activity in aged SMACre/β-cat mice were comparable, and eventually developed into pilomatricoma-like tumors/cysts over extended time frames (> 6 weeks) (Fig. S6E).

### Constitutive Wnt/β-catenin signaling induces squamous transdifferentiation of mammary epithelial cells

**Fig. 5:**
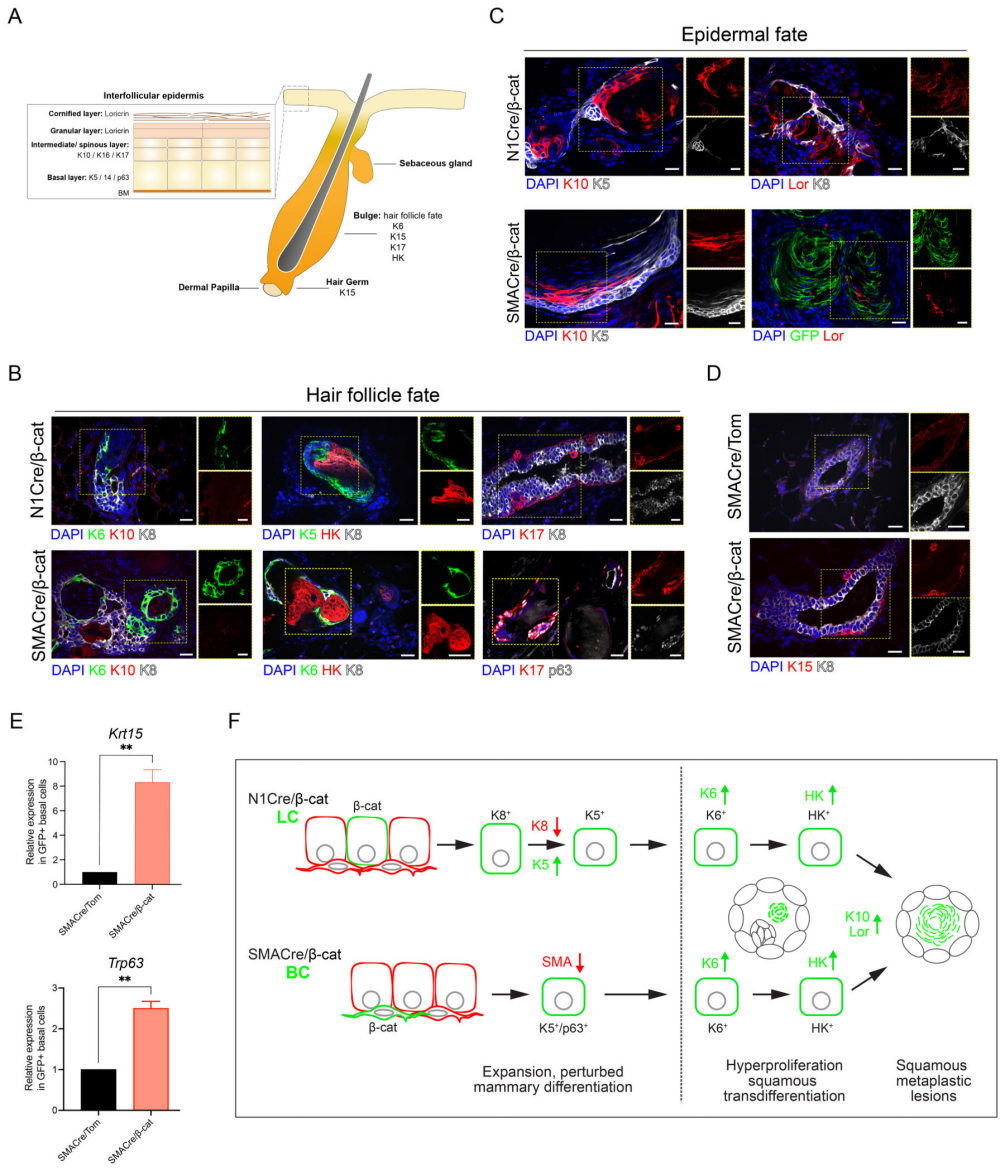
Constitutive Wnt/β-catenin signaling induces squamous transdifferentiation of both LC and BC mammary epithelial cells. **(A)** Scheme depicting the differentiation markers of adult hair follicle and interfollicular epidermis. **(B)** Representative sections of N1Cre/β-cat and SMACre/β-cat mammary glands showing acquired expression of the hair follicle markers K6 (left and center panels) and Hair Keratin (HK) (middle panels) and upregulation of K17 and p63 (right panels) in early lesions. **(C)** Representative sections of N1Cre/β-cat and SMACre/β-cat mammary glands showing acquired expression of the interfollicular epidermis markers K10 (left panels) and Loricrin (right panels) in advanced lesions. **(D)** Representative sections of SMACre/Tom and SMACre/β-cat mammary glands showing increased expression of the basal marker K15 in budding early lesions. Scale bar: 20 μm. **(E)** RT-qPCR analysis of *Krtl5* and *Trp63* expression in sorted GFP+ BC from SMACre/Tom and SMACre/β-cat glands. Graph shows mean ± SEM (p <0.01 Welch’s t-test; n=4 independent experiments). **(F)** Schematic representation of the timeline of squamous trans-differentiation induced by β-catenin stabilization in luminal (top) or basal (bottom) mammary epithelial cells.

Our longitudinal IVM studies and histological analysis of early mutant β-catenin-induced lesion development consistently revealed the formation of stereotypical bud-like clusters of epithelial cells (Fig.1I-L, Fig. 2M-N, S2B, S3H). These frequently displayed divergent upward displacement of epithelial nuclei towards newly forming growths, with epithelial cells organizing themselves into a compact arrangement (Fig.1I-L, Fig. 2M-N, S2B, S3H). This dynamic cellular rearrangement is reminiscent of early stage embryonic hair follicle formation (Devenport and Fuchs, 2008), and that observed in adult mouse skin in response to β-catenin activation in hair follicle stem cells, which drives new axes of hair follicle growth (Deschene et al., 2014). Moreover, long-term, non-lineage-specific targeting of stabilized β-catenin in mammary tissues using MMTV-Cre and WAP-Cre models was previously shown to induce squamous metaplasia, a process characterized by the expression of epidermal markers (Miyoshi et al., 2002b, 2002a). Our analysis also indicated that lesions arising in both N1 and SMACre models undergo squamous metaplasia, and the appearance of pilomatricoma-like tumors/cysts. Considering these similarities, we next investigated whether luminal or basal targeting of mutant β-catenin resulted in epidermal transdifferentiation by staining for different markers of intrafollicular epidermis (IFE) and hair follicles (HF) (Fig. 5A) (Plikus et al., 2012). Immunostaining for the HF marker K6, normally only present in TEBs at puberty (Grimm et al., 2006; Smith et al., 1990), revealed its widespread expression in early and late lesions in both N1Cre/β-cat and SMACre/β-cat models (Fig. 5B). Elevated K17 levels and ectopic hair keratin (HK) expression were also observed in early and late aberrant structures (Fig. 5B, S6F). Although undetectable at earlier stages (Fig. 5B, left panel), ectopic expression of the suprabasal skin marker K10 was observed in advanced basal- and luminal-derived lesions (Fig. 5C, left panel). Notably, mutant-induced lesions also acquired Loricrin expression, a highly specific marker for the cornified and granular IFE layers (Fig. 5C, right panel, S6F). Finally, mammary epithelial cells in SMACre/β-cat mice possessed elevated levels of K15 and p63 expression, also indicative of an increasingly epidermal-like state (Fig.5D-E). Collectively, our findings suggest that, regardless of the mammary lineage targeted, β-catenin stabilization drives the acquisition of a HF and epidermal differentiation program in mammary epithelial cells, resulting in squamous metaplasia (Fig. 5F).

## Discussion

Dysregulated Wnt/β-catenin activity is a hallmark of several types of cancer. Links between Wnt signaling and mammary tumorigenesis are well-established, yet its role in the initiation of different breast cancer subtypes remains poorly understood (van Schie and van Amerongen, 2020). The early molecular and cellular mechanisms underlying Wnt-driven mammary tumorigenesis are particularly unclear, largely due to a reliance on analyzing advanced tumors in previous studies. This is further hampered by discrepancies observed between available transgenic models of Wnt pathway activation, and the different mammary cell lineages targeted (Imbert et al., 2001; Miyoshi and Hennighausen, 2003; Miyoshi et al., 2002b, 2002a; Moumen et al., 2013; Teissedre et al., 2009; Teuliere et al., 2005). Moreover, until now, the impact of targeting a stabilized form of β-catenin from its endogenous promoter using *Catnb^+/lox(ex3)^* transgenic mice (Harada et al., 1999) to the mammary basal epithelial compartment, and how this compares to the phenotype observed when targeted to hormone responsive LC remained unexplored. Here, we sought to assess the phenotypes elicited by the same oncogenic β-catenin mutant on the *in situ* behavior of distinct mammary epithelial cell types, by coupling longitudinal IVM with lineage-specific activation of the Wnt/β-catenin pathway.

Real-time, *in vivo* imaging by IVM provides an essential window into the dynamic cellular events contributing to tissue development and pathology. This powerful approach has provided important insights into the growth, progression, metastasis and therapeutic responses of a plethora of cancer types, including breast cancer (Condeelis and Weissleder, 2010; Ellenbroek and Van Rheenen, 2014; Lloyd-Lewis, 2020). Yet, the application of IVM to study the earliest stages of neoplastic development remains largely absent, with most previous studies focused on imaging established tumors (Lloyd-Lewis, 2020). Here, we were able to capture the earliest cellular events underlying the impact of mutagenic Wnt/β-catenin signaling on the dynamics of distinct subpopulations of mammary epithelial cells *in vivo* and in real time, and its effects on the organization and differentiation of the mammary epithelium on a tissue scale. Interestingly, IVM revealed that constitutive stabilization of β-catenin from its endogenous promoter in either LC or BC caused epithelial cells to cluster in a compact, circular arrangement that either gave rise to ectopic bud-like growths or radially expanded over time. This was particularly evident when targeted to the basal mammary epithelial layer, where precocious budding could be readily visualized by day-to-day IVM. The precise mechanisms underlying these observations, and why some mutant cells were unable to give rise to new buds (e.g. mutant cap-in-body cells in TEBs), however, remains unclear. Nevertheless, the cellular behaviors revealed by IVM showed remarkable similarities to that observed during ectopic HF formation in response to β-catenin activation in HF stem cells (Deschene et al., 2014). Indeed, our data showed that these early, β-catenin-induced changes to epithelial cell organization and behavior reflects the progressive transdifferentiation of mammary cells to a hair follicle/epidermal-like fate, marked by the acquisition of HF and IFE marker expression (Fig. 5F). In support, analogous phenotypes were recently observed in mammary organoid cultures treated with high-dose CHIR99021, a potent activator of the Wnt/β-catenin pathway (Mourao et al., 2021). Based on our findings, we believe that constitutive Wnt activity in LC initially drives their conversion to basal-like cells, by repressing K8 expression and inducing basal traits such as K5/14, Id4 and p63 expression, and that they subsequently enter the same program of squamous transdifferentiation as mutant BC. This may underlie the observed differences in the timing and rate of keratinized lesion development in response to lineage-specific mutant β-catenin activity, with the appearance of squamous ‘rosette-like’ growths in the mammary epithelium of N1Cre/β-cat mice taking considerably longer as compared to SMACre/β-cat mice.

Our data, consistent with published work (Miyoshi et al., 2002b, 2002a), revealed that the aberrant mammary differentiation program induced by β-catenin activation leads to the formation of squamous metaplasia composed of keratinized “rosette-like’” structures and the appearance of ghost cells (characteristic of epidermal lesions and pilomatricoma tumors). These pilomatricoma-like lesions, comprised of basaloid proliferative cells which mature into structureless eosinophilic cells lacking nuclei, are more frequently observed when β-catenin activation is induced in adult mice and its effects are monitored over longer chase times (Fig. S5G and S6E). This is in contrast to alternative mouse models where N-terminally truncated β-catenin is ectopically expressed downstream of mammary promoters, which give rise to adenocarcinomas (Imbert et al., 2001; Michaelson and Leder, 2001; Teissedre et al., 2009; Teuliere et al., 2005). Discrepancies between the models may be explained by potential inherent differences between transgenic lines, in addition to differences in the potency of β-catenin activation. Indeed, levels of accumulated β-catenin driven by its endogenous promoter in the mammary glands of *Catnb^+/lox(ex3)^* transgenic mice (Harada et al., 1999) may be insufficient to drive adenocarcinoma development, compared to N-terminally truncated β-catenin transgenes driven by strong promoters such as a MMTV-LTR (Imbert et al., 2001; Michaelson and Leder, 2001; Miyoshi and Hennighausen, 2003).

In summary, by high-resolution longitudinal IVM, we visualized the dynamic cellular changes induced by lineage-specific mutant β-catenin accumulation *in situ* during the earliest stages of hyperplastic lesion development. We show that, regardless of the targeted cell of origin, aberrant β-catenin accumulation induces a hair-follicle/epidermal differentiation program in mammary epithelial cells, leading to the formation of squamous metaplasia. Importantly, our findings provide further evidence that the mammary epithelium is inherently predisposed towards acquiring an epidermal-like fate upon constitutive Wnt/β-catenin activation. As similar phenotypes are observed in other glandular tissues, including the prostate (Bierie et al., 2003), aberrant Wnt/β-catenin pathway activity is likely capable of over-riding existing differentiation program in several epithelial cell types to drive pathological tissue development.

### Limitations of the study

Our results indicate that aberrant β-catenin activation in LC or BC elicits a very similar phenotypic outcome, albeit with different dynamics. However, intrinsic differences in recombination efficiency between the two inducible Cre lines used in this study could also account for the different latencies in lesion development observed. Furthermore, mutant cells are identified by their expression of the GFP marker, which depends on an independent reporter transgene; therefore, caution should be applied when assuming that every GFP+ cells is mutant, in the absence of clear cytoplasmic/nuclear β-catenin immunostaining.

## Star Methods

### Resource Availability

#### Lead Contact

Further information and requests for resources and reagents should be directed to and will be fulfilled by the Lead Contact, Silvia Fre (silvia.fre@curie.fr).

#### Materials Availability

This study did not generate new unique reagents.

### Experimental model and subject details

#### Mouse models

All studies and procedures involving animals were in strict accordance with the recommendations of the European Community (2010/63/UE) for the Protection of Vertebrate Animals used for Experimental and other Scientific Purposes. Approval was provided by the ethics committee of the Institut Curie CEEA-IC #2021-029 and the French Ministry of Research (reference #34364-202112151422480). We comply with internationally established principles of replacement, reduction, and refinement in accordance with the Guide for the Care and Use of Laboratory Animals (NRC 2011). Husbandry, supply of animals, as well as maintenance and care in the Animal Facility of Institut Curie (facility license #C75–05–18) before and during experiments fully satisfied the animal’s needs and welfare. All animals were housed in individually ventilated cages under a 12:12 h light-dark cycle, with water and food available ad libitum. All mice were sacrificed by cervical dislocation.

All mouse lines (Mus musculus) used have been previously described and were of mixed genetic background. N1Cre^ERT2^ (Fre et al., 2011) and SMACre^ERT2^ (Wendling et al., 2009) were crossed to the double fluorescent reporter Rosa26^mT/mG^ (Muzumdar et al., 2007) and inducible mutant β-catenin *(Catnb^+/lox(ex3)^* (Harada et al., 1999) (kindly provided by Lionel Larue, Institut Curie) transgenic lines. We exclusively analyzed female mice and no randomization methods were performed. The age of the mice at the time of experiment is specified in the text, and included animals at the beginning of puberty (4-5 weeks of age) and the adult stage (up to 10 months of age). Reporter expression and β-catenin stabilization was induced in N1Cre^ERT2^ or SMACre^ERT2^ females by a single intraperitoneal injection of tamoxifen free base (Euromedex) prepared in sunflower oil containing 10% ethanol (0.1 mg per g of mouse body weight), unless indicated otherwise in figure legends. Using this dose, mammary gland development appeared to progress unabated, as previously reported (Rios et al., 2014). In experiments exceeding 8-10 days, TAM doses were reduced 10-fold (0.01mg per g of mouse body weight) to avoid potential systemic toxicity following long-term β-catenin stabilization. For EdU (5-ethynyl-2-deoxyuridine) labelling experiments, mice were injected intraperitoneally with 20 mg per kg of mouse body weight of EdU 2 h before harvesting mammary gland tissues.

### Method Details

#### Intravital imaging

Intravital imaging through a titanium/glass mammary imaging window were based on previously published protocols (Messal et al., 2021). Intravital imaging through our custom-made PDMS imaging windows was performed as previously described (Jacquemin et al., 2021). Briefly, mammary imaging windows were surgically implanted over the fourth mammary glands of 5-6-week-old mice at the indicated times after TAM administration. Mice were anaesthetized using isoflurane (1.5% isoflurane/medical air mixture) and placed in a facemask with a custom designed holder to stabilize windows during imaging acquisition. Imaging was performed on an upright Nikon A1R multiphoton microscope equipped with a Spectra-Physics Insight Deepsee laser, conventional and resonant scanners, GaAsP non-descanned detectors using 16x NA 0.8 or 25x NA 1.1 PlanApo LambdaS water objectives. An excitation wavelength of 960 nm was used for GFP and TdTomato, in addition to second harmonic generation (SHG) imaging of collagen. Mammary epithelial structures imaged in timelaspe were acquired every 30 min using a *Z*-step size of 2 μm. For long-term, longitudinal imaging z-stacks (with z-step size of 2 μm) of epithelial structures were taken either 1-2x daily for up to 8 days, or 2-3x weekly for up to 3 weeks (as indicated in figure legends).

#### Image processing and analysis

For high-resolution reconstruction of time-lapse and longitudinal acquisitions, regions of interest encompassing discrete Z-stack sizes were selected and registered using the StackReg Rigid Body plugin in FIJI (ImageJ v1.53) (Long et al., 2012; Schneider et al., 2012). Images were processed using a Gaussian blur filter (0.5-1 pxl radius) in FIJI (ImageJ v1.53). For the analysis of cap-in-body cell death, images of 6 TEBs from SMACre and SMACre/β-cat mice were randomly selected and the number of GFP+ cap-in-body basal cell clusters (typically encompassing between 1-5 cells/cluster) observed at the start of imaging (t0) and 24 hours later (t24) manually counted. For area and perimeter measurements of discrete cell clusters and lesions, images were processed using a Median filter (1-2px) and segmented in the GFP channel using Otsu threshold, followed by Distance Transform Watershed (Borgefors) and analysis function in the MorpholibJ plugin (Legland et al., 2016) in FIJI (ImageJ v1.53). Generated masks were manually checked against raw data for consistency prior to extracting area and perimeter measurements. Only discrete lesions were measured over time i.e. no further measurements were taken in growing lesions that had collided/fused with a neighbouring lesion. For nuclear masks, DAPI images were processed with a median filter (1-2px) and an inverted LUT applied, with nuclei outlined manually using the freehand selection tool in FIJI (ImageJ v1.53). For generating membrane GFP masks, images were processed using the Top Hat filter in ImageJ, and an inverted LUT applied prior to segmentation using the Morphological Segmentation function in MorpholibJ (Legland et al., 2016) in FIJI (ImageJ v1.53).

#### Optical tissue clearing and whole-mount immunostaining

Mammary gland pairs 2 or 3 were dissected, spread on TetraPak card and optimally fixed for 6-9 h in 10% Neutral Buffered Formalin (NBF) at room temperature. Mammary glands were cut into large pieces (~15×15×2 mm) for immunostaining and tissue clearing, as previously described in detail (Lloyd-Lewis et al., 2016). Optical tissue clearing was performed using a modified CUBIC (Reagent 1A) protocol (Susaki and Ueda, 2016). Briefly, tissues were immersed in CUBIC Reagent 1A (urea (10% w/w), Quadrol® (5% w/w), triton X-100 (10% w/w), NaCl (25 mM) in distilled water) for 2-3 days at 37°C, washed in PBS and blocked overnight at 4°C in PBS containing normal goat serum (10%) and triton X-100 (0.5%). Tissue was incubated in primary antibodies diluted in blocking buffer at 4°C for 4 days with gentle agitation. Tissue was washed in PBS (3 x 1 h) and incubated with secondary Alexa-fluor conjugated antibodies at 4°C for 2 days with gentle agitation before further washing in PBS (3 x 1h) and incubation with DAPI (10 μM) for 2-3 h at room temperature. Tissues were imaged in CUBIC Reagent 2.

#### Immunohistochemistry

IHC was performed according to a previously published protocol (Sargeant et al., 2014). Briefly, formalin-fixed paraffin embedded 5-7 μm mammary tissue sections were deparaffinized in xylene and rehydrated in a reducing ethanol series. Tissue was permeabilized in phosphate buffered saline (PBS) containing triton X-100 (0.5%). Heat-induced epitope retrieval was performed for 11 min at 110°C in Tris 10mM EDTA 1mM (pH 9) for β-catenin immunostaining or in sodium citrate (0.01 M, pH 6) for all other antigens. Slides were blocked in PBS containing fetal bovine serum (5%) BSA (2%) and triton x-100 (0.25%) for 1 h. Primary antibodies diluted in blocking buffer were incubated overnight at 4°C in a humidified chamber. Secondary antibodies diluted in PBS were incubated for 1 h at room temperature. EdU detection was performed using the Click-iT EdU Alexa Fluor 647 Imaging Kit (Molecular Probes), according to the manufacturer’s instructions. Nuclei were stained with DAPI dilactate (625 ng/mL) for 10-15 min at room temperature or, in the case of Edu detection, with Hoechst33342 10 μg/ml for 30 min at room temperature. Slides were mounted using Aqua-Polymount. TUNEL assays were performed using TdT digoxygenin nick-end labeling with Apoptag Plus (Sigma Aldrich) following manufacturer’s instructions. Nuclei were counterstained with methyl green.

#### Fluorescence confocal microscopy of fixed tissues

##### 3D imaging

CUBIC-cleared tissues were imaged in CUBIC Reagent 2 in 35 mm glass-bottom Fluoro-dishes. Images were acquired using an LSM780 or LSM880 inverted laser scanning confocal microscope (Carl Zeiss) equipped with 10×/0.3 PL NEOFLUAR or 25×/0.8 LD LCI PLAN-APO objective lenses. For standard 4-color imaging, laser power and gain were adjusted manually to give optimal fluorescence for each fluorophore with minimal photobleaching. Imaging depths were recorded from the top of the epithelial structure being imaged (typically ~350 μm through the native fat pad). Image reconstructions were generated in FIJI (ImageJ v1.53) using the Bio-Formats plugin (National Institutes of Health) (Linkert et al., 2010; Schindelin et al., 2012). Denoising of 3D image stacks was performed in MATLAB (R2014a, The Mathoworks Inc.) (Boulanger et al., 2010).

##### 2D imaging

Images of stained sections were acquired using an upright spinning disk (CSU-X1 scan-head from Yokogawa) confocal microscope (Carl Zeiss, Roper Scientific France), equipped with a CoolSnap HQ2 charge coupled device (CCD) camera (Photometrics) and PLAN APO ×63/1.4 NA and PLAN APO 40x/1.3NA objective lenses. Images were captured using Metamorph and processed in FIJI (ImageJ v1.53).

#### Mammary gland dissociation and Flow Cytometry

Single cell dissociation was performed through enzymatic digestion with 600 U ml^-1^ collagenase (Sigma) and 200 U ml^-1^ hyaluronidase (Sigma) for 90 min at 37 °C. Cells were further dissociated in TrypLE (Gibco) for 3 min, in 5 mg ml^-1^ dispase (Roche) and 0.1 mg ml^-1^ DNase I (Sigma) for 5 min, and then in 0.63% NH_4_Cl and filtered through a 40 μm cell strainer to obtain a single cell preparation for FACS. Cell labelling and flow cytometry were performed as described previously (Koren et al., 2015) using LSRII or FACS ARIA flow cytometers (BD). Dead cells (DAPI^+^), and CD45^+^/CD31^+^/Ter119^+^ (Lin^+^) non-epithelial cells were excluded. The following antibodies were all purchased from Biolegend and were used at a 1:100 final concentration: PE/Cy7 anti-mouse CD24, PE/Cy7 anti-mouse Epcam, AlexaFluor700 anti-mouse/rat CD29, APC/Cy7 anti-mouse CD49f, lineage markers: APC anti-mouse CD31, APC anti-mouse Ter119, APC anti-mouse CD45, isotype controls: PE rat IgM, PerCP/Cy5.5 rat IgGa, PE/Cy7 rat IgG2a, APC/Cy7 rat IgG2a, APC rat IgG2b. The purity of sorted populations was approximately 95%. The results were analyzed using FlowJo software (v10, BD).

#### Colony forming assays

Sorted cells well cultured on irradiated 3T3 cell feeders in 24-well plates for 7 days. Luminal cells were plated at a density of 400 cells per well and cultured in DMEM/F12 medium containing fetal bovine serum (10%), 5 μg/ml insulin (Sigma-Aldrich), 10 ng/ml EGF (Invitrogen, Life Technologies) and 100 ng/ml cholera toxin (ICN Biochemicals). Basal cells were plated at a density of 1500 cells per well and cultured in DMEM/F12 medium containing fetal bovine serum (1%), 1/50 diluted B27 supplement (ThermoFisher), 5 μg/ml insulin (Sigma-Aldrich), 10 ng/ml EGF (Invitrogen, Life Technologies). Colonies were fixed in 4% PFA and stained with hematoxylin/eosin and pictures were acquired using a Leica MZ8 binocular. To evaluate colony number and size, ImageJ software was used. For proliferation analysis, colonies were incubated with EdU at 2 μg/ml for 1 h prior to PFA fixation. Edu detection was performed with Click-iT EdU Alexa Fluor 647 Imaging Kit (Molecular Probes), according to the manufacturer’s instructions.

#### RNA extraction and RT-qPCR

RNA was extracted from sorted cells with the RNeasy Microkit including DNAse treatment (Qiagen) and reverse-transcribed using SuperScript Vilo IV Reverse-Transcriptase (Invitrogen). Quantitative PCR was performed using the QuantiNova SYBR Green PCR kit (Qiagen) on a LightCycler 480 real-time PCR system (Roche). Relative gene expression was determined by normalizing to reference genes (*Gapdh* and *18s*) using the comparative CT method. The primers used for amplification were designed using Oligo 6.8 software (Molecular Biology Insights) and synthesized by Eurogentec (Table S3). Other primers were purchased from Qiagen.

#### Single molecule RNA Fluorescence In Situ Hybridization (smRNA FISH)

smRNA FISH was performed on mouse PFA-fixed paraffin sections using RNAscope® Multiplex Fluorescent Detection Kit v2 kit and pipeline following manufacturer’s recommendations. Axin2 mRNA were labelled using RNAscope® Probe Mm-Axin2-C3. In order to subsequently perform immunostaining after the FISH, a protease III step not exceeding 20 minutes was included. Subsequent antibody staining was performed as described above.

#### Quantification and Statistical Analysis

Experiments were performed in biological replicates as stated in figure legends. For each experiment, we have used at least n=3 animals, and experiments with at least n=3 replicates were used to calculate the statistical value of each analysis. Data processing and statistical analysis were performed in Prism (v9.2, GraphPad). All graphs show mean ± SEM. Statistical analysis was performed with two-tailed unpaired Welch’s t-tests, unless otherwise stated in figure legends.

## Supplementary Material

Movie S1 **Supplemental Movie 1:** Longitudinal intravital imaging of a mammary end bud structure in a pubertal N1Cre/Tom mouse showing the dynamic cellular rearrangements and behavior of GFP+ luminal epithelial cells over 6 days (144 h). Red: non-recombined membrane tdTomato-expressing mammary epithelial cells; cyan: collagen (SHG). Related to Figure 1.

Movie S2 **Supplemental Movie 2:** Acute time-lapse IVM of GFP+ luminal epithelial cells residing in a mammary end bud structure of a pubertal N1Cre/Tom mouse. Red: non-recombined membrane tdTomato-expressing mammary epithelial cells; cyan: collagen (SHG). Total movie length is 06:00 (h:min). Related to Figure 1.

Movie S3 **Supplemental Movie 3:** Acute time-lapse IVM of GFP+ luminal epithelial cells in the mammary gland of a pubertal N1Cre/β-cat mouse. Red: non-recombined membrane tdTomato-expressing mammary epithelial cells. Total movie length is 16:30 (h:min). Related to Fig. 1 and S2.

Movie S4 **Supplemental Movie 4:** Acute time-lapse IVM of GFP+ luminal epithelial cells in the mammary gland of a pubertal N1Cre/β-cat mouse. Red: non-recombined membrane tdTomato-expressing mammary epithelial cells. Total movie length is 16:00 (h:min). Related to Fig. 1 and S2.

Movie S5 **Supplemental Movie 5:** Acute time-lapse IVM of GFP+ basal epithelial cells in the mammary gland of a pubertal SMACre/β-cat mouse. Red: non-recombined membrane tdTomato-expressing mammary epithelial cells. Total movie length is 12:30 (h:min). Related to Figure 2.

Movie S6 **Supplemental Movie 6:** Longitudinal intravital imaging of a mammary end bud structure in a pubertal SMACre/β-cat mouse revealing the expansion of mutant GFP+ cap-in-body epithelial cells over 9 days (216 h). Red: non-recombined membrane tdTomato-expressing mammary epithelial cells; cyan: collagen (SHG). Related to Figure 2.

Supplementary Figures

## Data Availability

All data reported in this paper will be shared by the lead contact upon request. This paper does not report original code. Any additional information required to reanalyze the data reported in this paper is available from the lead contact upon request.

## References

[R1] Bierie B, Nozawa M, Renou JP, Shillingford JM, Morgan F, Oka T, Taketo MM, Cardiff RD, Miyoshi K, Wagner KU (2003). Activation of β-catenin in prostate epithelium induces hyperplasias and squamous transdifferentiation. Oncogene.

[R2] Boulanger J, Kervrann C, Bouthemy P, Elbau P, Sibarita J-B, Salamero J (2010). Patch-based nonlocal functional for denoising fluorescence microscopy image sequences. IEEE Trans Med Imaging.

[R3] Bouras T, Pal B, Vaillant F, Harburg G, Asselin-Labat M-L, Oakes SR, Lindeman GJ, Visvader JE (2008). Notch signaling regulates mammary stem cell function and luminal cell-fate commitment. Cell Stem Cell.

[R4] Condeelis J, Weissleder R (2010). In vivo imaging in cancer. Cold Spring Harb Perspect Biol.

[R5] Corominas-Murtra B, Scheele CLGJ, Kishi K, Ellenbroek SIJ, Simons BD, van Rheenen J, Hannezo E, van Rheenen J, Hannezo E (2020). Stem cell lineage survival as a noisy competition for niche access. Proc Natl Acad Sci.

[R6] Dawson CA, Mueller SN, Lindeman GJ, Rios AC, Visvader JE (2021). Intravital microscopy of dynamic single-cell behavior in mouse mammary tissue. Nat Protoc.

[R7] Deschene ER, Myung P, Rompolas P, Zito G, Sun TY, Taketo MM, Saotome I, Greco V (2014). β-Catenin activation regulates tissue growth non-cell autonomously in the hair stem cell niche. Science (80-).

[R8] Devenport D, Fuchs E (2008). Planar polarization in embryonic epidermis orchestrates global asymmetric morphogenesis of hair follicles. Nat Cell Biol.

[R9] Ellenbroek SIJ, Van Rheenen J (2014). Imaging hallmarks of cancer in living mice. Nat Rev Cancer.

[R10] Ewald AJ, Huebner RJ, Palsdottir H, Lee JK, Perez MJ, Jorgens DM, Tauscher AN, Cheung KJ, Werb Z, Auer M (2012). Mammary collective cell migration involves transient loss of epithelial features and individual cell migration within the epithelium. J Cell Sci.

[R11] Fre S, Hannezo E, Sale S, Huyghe M, Lafkas D, Kissel H, Louvi A, Greve J, Louvard D, Artavanis-Tsakonas S (2011). Notch Lineages and Activity in Intestinal Stem Cells Determined by a New Set of Knock-In Mice. PLoS One.

[R12] Grimm SL, Bu W, Longley MA, Roop DR, Li Y, Rosen JM (2006). Keratin 6 is not essential for mammary gland development. Breast Cancer Res.

[R13] Harada N, Tamai Y, Ishikawa T-O, Sauer B, Takaku K, Oshima M, Taketo MM (1999). Intestinal polyposis in mice with a dominant stable mutation of the β-catenin gene. EMBO J.

[R14] Imbert A, Eelkema R, Jordan S, Feiner H, Cowin P (2001). ΔN89β-catenin induces precocious development, differentiation, neoplasia in mammary gland. J Cell Biol.

[R15] Incassati A, Chandramouli A, Eelkema R, Cowin P (2010). Key signaling nodes in mammary gland development and cancer: β-catenin. Breast Cancer Res.

[R16] Jacquemin G, Benavente-Diaz M, Djaber S, Bore A, Dangles-Marie V, Surdez D, Tajbakhsh S, Fre S, Lloyd-Lewis B (2021). Longitudinal high-resolution imaging through a flexible intravital imaging window. Sci Adv.

[R17] Jardé T, Dale T (2012). Wnt signalling in murine postnatal mammary gland development. Acta Physiol.

[R18] Jardé T, Lloyd-Lewis B, Thomas M, Kendrick H, Melchor L, Bougaret L, Watson PDPD, Ewan K, Smalley MJMJ, Dale TCTC (2016). Wnt and Neuregulin1/ErbB signalling extends 3D culture of hormone responsive mammary organoids. Nat Commun.

[R19] Koren S, Reavie L, Couto JP, De Silva D, Stadler MB, Roloff T, Britschgi A, Eichlisberger T, Kohler H, Aina O (2015). PIK3CAH1047R induces multipotency and multi-lineage mammary tumours. Nature.

[R20] Lafkas D, Rodilla V, Huyghe M, Mourao L, Kiaris H, Fre S (2013). Notch3 marks clonogenic mammary luminal progenitor cells in vivo. J Cell Biol.

[R21] Legland D, Arganda-Carreras I, Andrey P (2016). MorphoLibJ: integrated library and plugins for mathematical morphology with ImageJ. Bioinformatics.

[R22] Li Y, Welm B, Podsypanina K, Huang S, Chamorro M, Zhang X, Rowlands T, Egeblad M, Cowin P, Werb Z (2003). Evidence that transgenes encoding components of the Wnt signaling pathway preferentially induce mammary cancers from progenitor cells.

[R23] Lilja AM, Rodilla V, Huyghe M, Hannezo E, Landragin C, Renaud O, Leroy O, Rulands S, Simons BD, Fre S (2018). Clonal analysis of Notch1-expressing cells reveals the existence of unipotent stem cells that retain long-term plasticity in the embryonic mammary gland. Nat Cell Biol.

[R24] Lim E, Vaillant F, Wu D, Forrest NC, Pal B, Hart AH, Asselin-Labat ML, Gyorki DE, Ward T, Partanen A (2009). Aberrant luminal progenitors as the candidate target population for basal tumor development in BRCA1 mutation carriers. Nat Med.

[R25] Linkert M, Rueden CT, Allan C, Burel JM, Moore W, Patterson A, Loranger B, Moore J, Neves C, MacDonald D (2010). Metadata matters: access to image data in the real world. J Cell Biol.

[R26] Liu BY, McDermott SP, Khwaja SS, Alexander CM (2004). The transforming activity of Wnt effectors correlates with their ability to induce the accumulation of mammary progenitor cells. Proc Natl Acad Sci U S A.

[R27] Lloyd-Lewis B (2020). Multidimensional Imaging of Mammary Gland Development: A Window Into Breast Form and Function. Front Cell Dev Biol.

[R28] Lloyd-Lewis B, Davis FM, Harris OB, Hitchcock JR, Lourenco FC, Pasche M, Watson CJ (2016). Imaging the mammary gland and mammary tumours in 3D: optical tissue clearing and immunofluorescence methods. Breast Cancer Res.

[R29] Long F, Zhou J, Peng H (2012). Visualization and Analysis of 3D Microscopic Images. PLoS Comput Biol.

[R30] MacDonald BT, Tamai K, He X (2009). Wnt/β-Catenin Signaling: Components, Mechanisms, and Diseases. Dev Cell.

[R31] Messal HA, van Rheenen J, Scheele CLGJ (2021). An Intravital Microscopy Toolbox to Study Mammary Gland Dynamics from Cellular Level to Organ Scale. J Mammary Gland Biol Neoplasia.

[R32] Michaelson JS, Leder P (2001). β-catenin is a downstream effector of Wnt-mediated tumorigenesis in the mammary gland. Oncogene.

[R33] Miyoshi K, Hennighausen L (2003). β-Catenin: A transforming actor on many stages. Breast Cancer Res.

[R34] Miyoshi K, Shillingford JM, Le Provost F, Gounari F, Bronson R, Von Boehmer H, Taketo MM, Cardiff RD, Hennighausen L, Khazaie K (2002a). Activation of β-catenin signaling in differentiated mammary secretory cells induces transdifferentiation into epidermis and squamous metaplasias. Proc Natl Acad Sci U S A.

[R35] Miyoshi K, Rosner A, Nozawa M, Byrd C, Morgan F, Landesman-Bollag E, Xu X, Seldin DC, Schmidt EV, Taketo MM (2002b). Activation of different Wnt/β-catenin signaling components in mammary epithelium induces transdifferentiation and the formation of pilar tumors. Oncogene.

[R36] Molyneux G, Geyer FC, Magnay F-A, McCarthy A, Kendrick H, Natrajan R, Mackay A, Grigoriadis A, Tutt A, Ashworth A (2010). BRCA1 basal-like breast cancers originate from luminal epithelial progenitors and not from basal stem cells. Cell Stem Cell.

[R37] Moumen M, Chiche A, Decraene C, Petit V, Gandarillas A, Deugnier MA, Glukhova MA, Faraldo MM (2013). Myc is required for β-catenin-mediated mammary stem cell amplification and tumorigenesis. Mol Cancer.

[R60] Mourao L, Zeeman AL, Wiese KE, Bongaarts A, Oudejans LL, Martinez IM, van de Grift YBC, Jonkers J, van Amerongen R (2021). Hyperactive WNT/CTNNB1 signaling induces a competing cell proliferation and epidermal differentiation response in the mouse mammary epithelium. bioRxiv.

[R38] Muzumdar MD, Tasic B, Miyamichi K, Li L, Luo L (2007). A global double-fluorescent Cre reporter mouse. Genesis.

[R39] Nusse R, Clevers H (2017). Leading Edge Review Wnt/b-Catenin Signaling, Disease, and Emerging Therapeutic Modalities.

[R40] Nusse R, Varmus HE (1982). Many tumors induced by the mouse mammary tumor virus contain a provirus integrated in the same region of the host genome. Cell.

[R41] Pittius CW, Sankaran L, Topper YJ, Hennighausen L (1988). Comparison of the Regulation of the Whey Acidic Protein Gene with that of a Hybrid Gene Containing the Whey Acidic Protein Gene Promoter in Transgenic Mice. Mol Endocrinol.

[R42] Plikus MV, Gay DL, Treffeisen E, Wang A, Supapannachart RJ, Cotsarelis G (2012). Epithelial stem cells and implications for wound repair. Semin Cell Dev Biol.

[R43] Rios AC, Fu NY, Lindeman GJ, Visvader JE (2014). In situ identification of bipotent stem cells in the mammary gland. Nature.

[R44] Rodilla V, Dasti A, Huyghe M, Lafkas D, Laurent C, Reyal F, Fre S (2015). Luminal Progenitors Restrict Their Lineage Potential during Mammary Gland Development. PLOS Biol.

[R45] Sargeant TJ, Lloyd-Lewis B, Resemann HK, Ramos-Montoya A, Skepper J, Watson CJ (2014). Stat3 controls cell death during mammary gland involution by regulating uptake of milk fat globules and lysosomal membrane permeabilization. Nat Cell Biol.

[R46] Scheele CLGJ, Hannezo E, Muraro MJ, Zomer A, Langedijk NSM, van Oudenaarden A, Simons BD, van Rheenen J (2017). Identity and dynamics of mammary stem cells during branching morphogenesis. Nature.

[R47] van Schie EH, van Amerongen R (2020). Aberrant WNT/CTNNB1 Signaling as a Therapeutic Target in Human Breast Cancer: Weighing the Evidence. Front Cell Dev Biol.

[R48] Schindelin J, Arganda-Carreras I, Frise E, Kaynig V, Longair M, Pietzsch T, Preibisch S, Rueden C, Saalfeld S, Schmid B (2012). Fiji: an open-source platform for biological-image analysis. Nat Methods.

[R49] Schneider CA, Rasband WS, Eliceiri KW (2012). NIH Image to ImageJ: 25 years of image analysis. Nat Methods.

[R50] Sleeman KE, Kendrick H, Robertson D, Isacke CM, Ashworth A, Smalley MJ (2007). Dissociation of estrogen receptor expression and in vivo stem cell activity in the mammary gland. J Cell Biol.

[R51] Smith GH, Mehrel T, Roop DR (1990). Differential keratin gene expression in developing, differentiating, preneoplastic, and neoplastic mouse mammary epithelium. Cell Growth Differ.

[R52] Steinhart Z, Angers S (2018). Wnt signaling in development and tissue homeostasis. Development.

[R53] Susaki EA, Ueda HR (2016). Whole-body and Whole-Organ Clearing and Imaging Techniques with Single-Cell Resolution: Toward Organism-Level Systems Biology in Mammals. Cell Chem Biol.

[R54] Teissedre B, Pinderhughes A, Incassati A, Hatsell SJ, Hiremath M (2009). MMTV-Wnt1 and-DN89b-Catenin Induce Canonical Signaling in Distinct Progenitors and Differentially Activate Hedgehog Signaling within Mammary Tumors. PLoS One.

[R55] Teuliere J, Faraldo MM, Deugnier MA, Shtutman M, Ben-Ze’ev A, Thiery JP, Glukhova MA (2005). Targeted activation of beta-catenin signaling in basal mammary epithelial cells affects mammary development and leads to hyperplasia. Development.

[R56] Tsukamoto AS, Grosschedl R, Guzman RC, Parslow T, Varmus HE (1988). Expression of the int-1 gene in transgenic mice is associated with mammary gland hyperplasia and adenocarcinomas in male and female mice. Cell.

[R57] Wendling O, Bornert J-M, Chambon P, Metzger D (2009). Efficient temporally-controlled targeted mutagenesis in smooth muscle cells of the adult mouse. Genesis.

[R58] Yu QC, Verheyen EM, Zeng YA (2016). Mammary development and breast cancer: A Wnt perspective. Cancers (Basel).

[R59] Zomer A, Ellenbroek SIJ, Ritsma L, Beerling E, Vrisekoop N, Van Rheenen J (2013). Brief Report: Intravital Imaging of Cancer Stem Cell Plasticity in Mammary Tumors. Stem Cells.

