# Supplementary material for: In vivo imaging of mammary epithelial cell dynamics in response to lineage-biased Wnt/β-catenin activation
Source: Cell Rep. Author manuscript; Available in PMC 2023 Oct 9. (PMC7615182; doi:10.1016/j.celrep.2022.110461)
Supplement: Supplementary Figures [file EMS188911-supplement-Supplementary_Figures.pdf]

Supplementary Figure 1

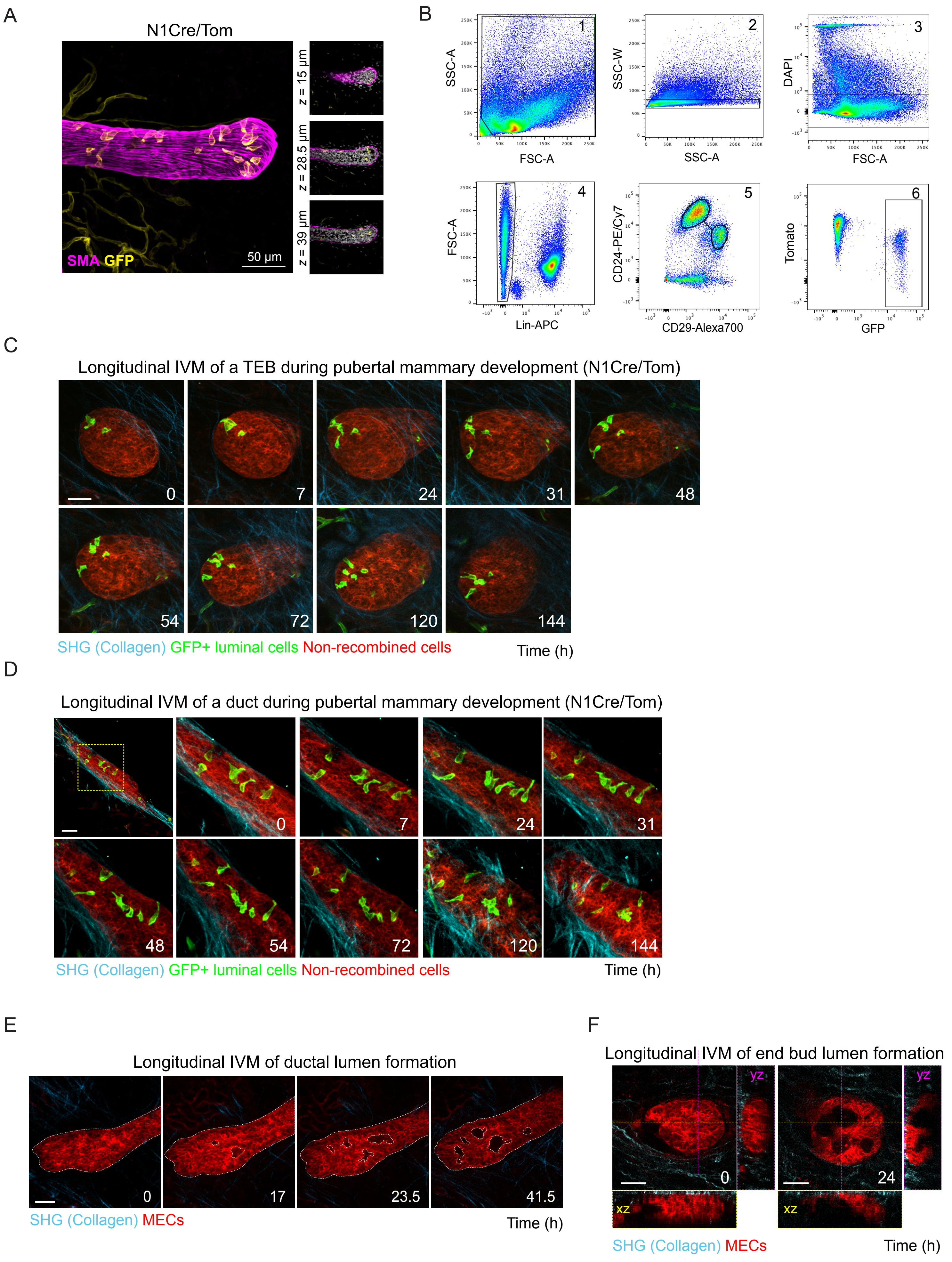

Fig. S1. Longitudinal IVM of wildtype luminal cell behavior during pubertal ductal development. Related to Fig. 1.

(A) Maximum intensity z-projection and thin optical slices (depth in *z* relative to the first image in the image sequence) of cleared mammary tissue from N1Cre/Tom mice immunostained with smooth muscle actin (SMA in pink). GFP+ cells (shown in yellow) are restricted to the luminal compartment < 1 week after low-dose tamoxifen administration. Scale bar: 50μm. **(B)** Representative dot plots of the applied FACS gating strategy. (1) FCS/SSC gating allows cell debris to be discarded, (2) SSC-A/SSC-W selects single cells, (3) DAPI exclusion selects live cells, (4) Lin exclusion (CD45/CD31/Ter119 cell surface marker proteins) eliminates hematopoietic and endothelial cells, (5) CD29/CD24 cell surface markers are used to identify Mammary Epithelial Cells (MEC) and (6) GFP/Tomato selects fluorescent cells. **(C-D)** IVM images of luminal GFP+ (green) mammary cells in a mammary end bud structure (C) or in a subtending duct (D) of a pubertal N1Cre/Tom mouse over time. Close up images included in Figure 1F, related to Supplemental Movie 1. **(E)** IVM images of a mammary ductal structure showing the process of lumen formation. **(F)** Longitudinal intravital images of a mammary TEB showing rapid lumen formation. Orthogonal views show *XZ* (*yellow line and box*) and *YZ* (*purple line and box*) planes. Red: non-recombined membrane tdTomato-expressing mammary epithelial cells; cyan: collagen (SHG). All scale bars: 50μm.

Supplementary Figure 2

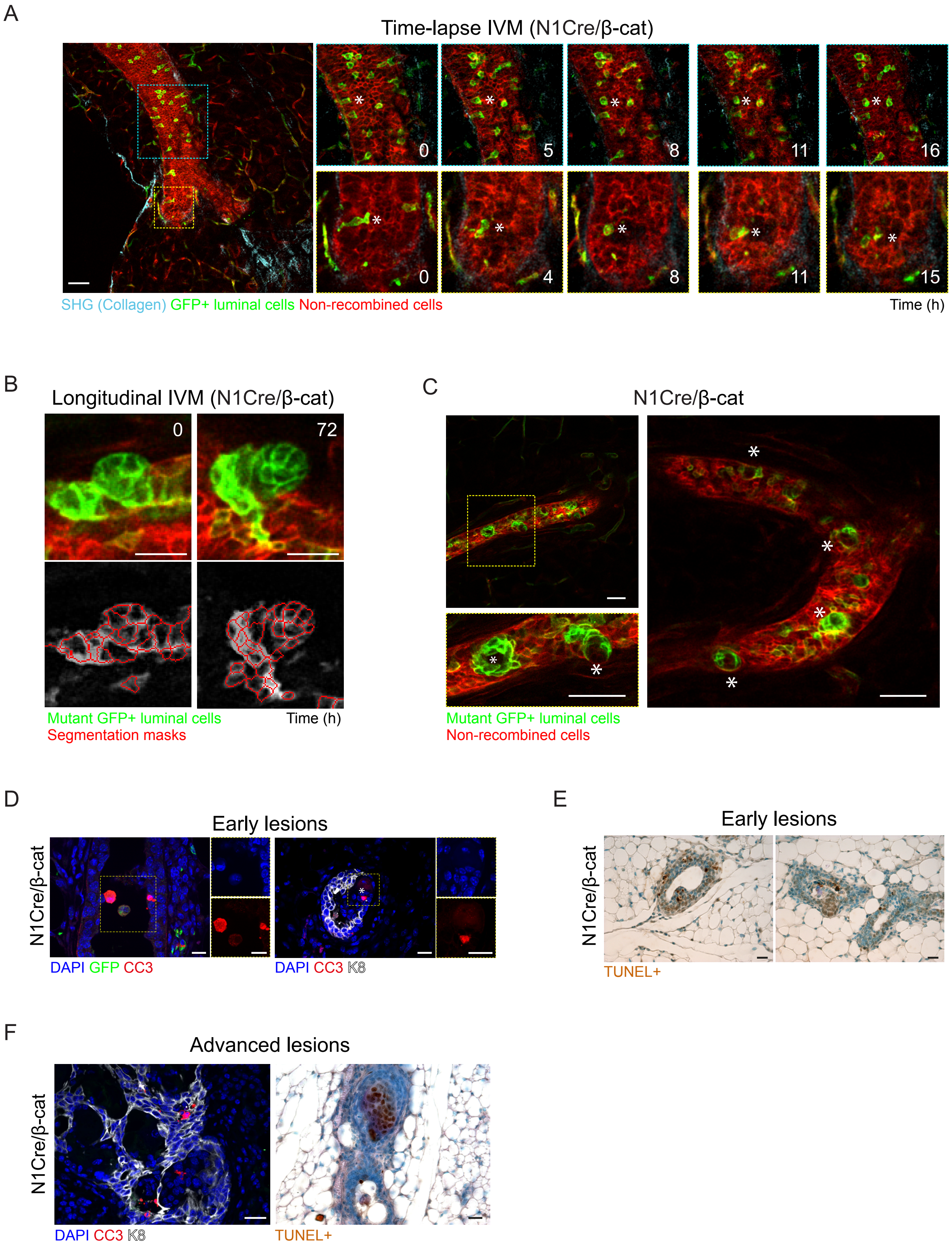

Fig. S2. Time-lapse and longitudinal intravital imaging of mutant β-cat luminal cells. Related to Fig. 1.

(A) Acute time-lapse IVM of luminal GFP+ (green) cell behavior in a mammary duct of a pubertal N1Cre/β-cat mouse performed 48 h after low-dose tamoxifen administration. Red: non-recombined membrane tdTomato-expressing mammary epithelial cells; cyan: collagen (SHG). Related to Supplemental Movies 3 and 4. Asterisks denote cells being tracked over time. Scale bar: 50 µm. **(B)** Segmentation masks of mutant GFP+ cells (green) in a developing lesion imaged over time by IVM (Region 2, Fig.1I). Scale bar: 20µm. **(C)** IVM images (single *z*-planes) of luminal GFP+ lesions in a mammary duct of a N1Cre/β-cat mouse 19 (left panel) and 21 (right panel) days after low-dose tamoxifen administration. Asterisks demark loss of fluorescence in the center of ring-like lesions. Scale bars: 50 µm. **(D)** Representative sections of N1Cre/β-cat mammary glands showing cleaved-caspase 3 (CC3) immunostaining in ductal and terminal end bud (TEB) regions, including within a ring-like cell cluster (asterisk, right panel). Scale bar: 20 µm. **(E)** TUNEL staining (brown) in N1Cre/β-cat mammary sections indicating that cells within early hyperplastic lesions undergo cell death. Nuclei are counterstained with Methyl green. Scale bar: 20 µm. **(F)** CC3 (in red, left) and TUNEL (in brown, right) staining in advanced lesions in N1Cre/β-cat mammary sections. DNA is stained by DAPI in blue (left panel); nuclei are counterstained with Methyl green in the right panel. Scale bar: 20 µm.

Supplementary Figure 3

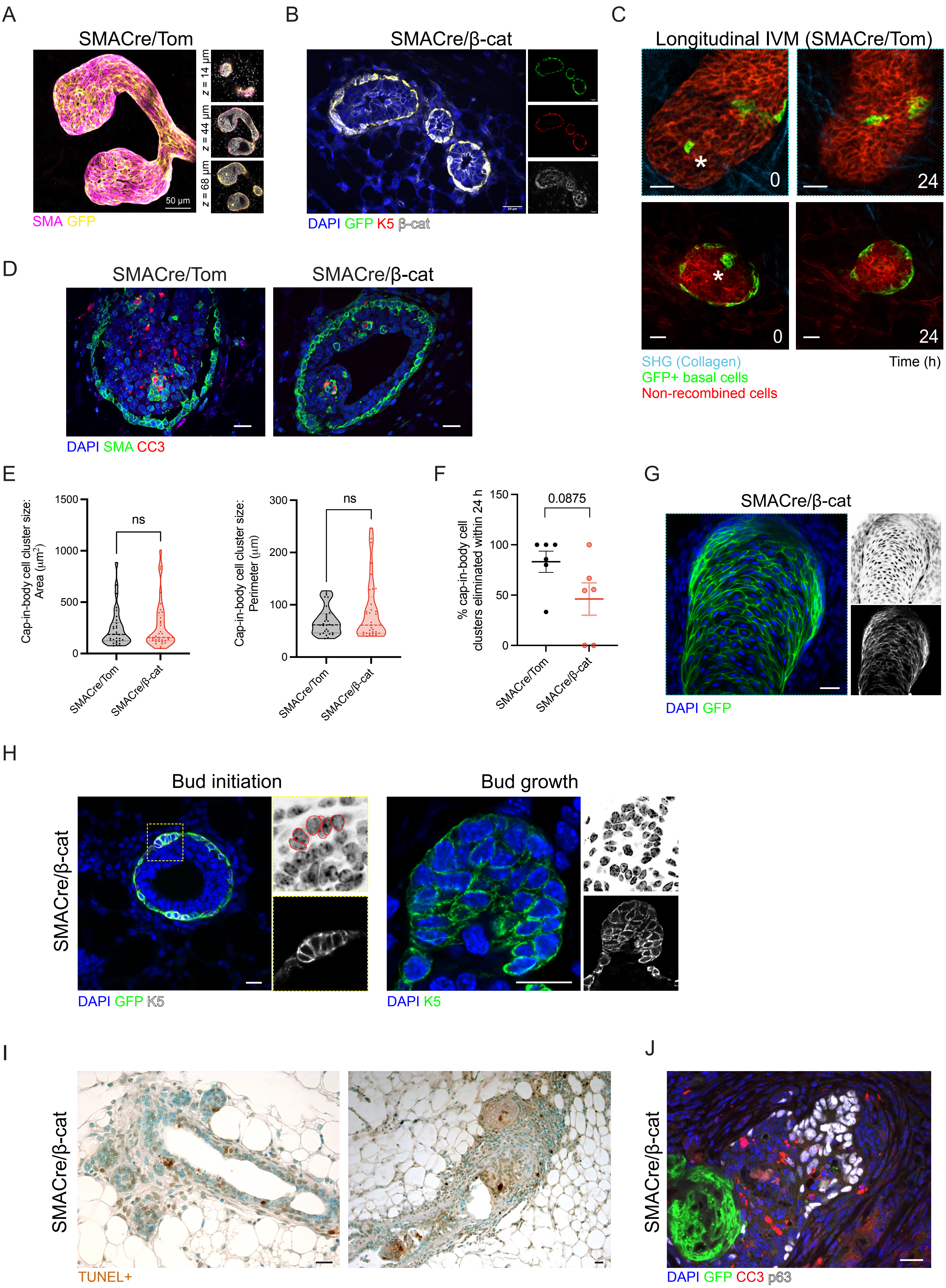

Fig. S3. Longitudinal IVM of wild-type and mutant β-cat basal cell behavior during pubertal ductal development. Related to Fig. 2.

(A) Maximum intensity z-projection and thin optical slices (depth in *z* relative to the first image in the image sequence) of cleared mammary tissue from SMACre/Tom mice immunostained with smooth muscle actin (SMA in pink). Recombined cells (shown in yellow) are restricted to the basal compartment. **(B)** Representative section of mammary tissue showing β-catenin accumulation in K5-expressing basal cells (in red) in SMACre/β-cat mice, coinciding with membrane GFP expression (in green). Anti-β-catenin staining is in white and DAPI labels nuclei in blue. **(C)** IVM images of mammary terminal end bud (TEB) structures in SMACre/Tom mice showing the elimination of basal GFP+ (green) cap-in-body cells within 24 h. Top panel: inset of Fig. S4A (blue box). Red: non-recombined membrane tdTomato-expressing mammary epithelial cells; cyan: collagen (SHG). Scale bar: 25 μm. **(D)** Representative sections of mammary gland tissues from pubertal SMACre/Tom and SMACre/β-cat mice showing cleaved-caspase 3 (CC3, red) immunostaining in terminal end buds (TEB). SMA expression (green) marks internalized cap-in-body basal cells. Scale bar: 20 μm. **(E)** Violin plots showing no statistical differences in the area (left) and perimeter (right) of GFP+ cap-in-body cell clusters in TEBs in mammary tissues in pubertal SMACre/Tom and SMACre/β-cat mice at the start of intravital imaging (t0). Lines depict the median and first and third quartile. n=30-33 clusters analyzed in 6 different TEBs from 4-5 mice per group. On average, TEBs in SMACre/Tom and SMACre/β-cat mice possessed 5 and 5.5 GFP+ clusters per TEB respectively (ns: not significant, Mann-Whitney test). **(F)** Graph showing the percentage of GFP+ cap-in-body cell clusters eliminated within 24 h in mammary TEBs of SMACre/Tom (average:83%) and SMACre/β-cat (average:46%) pubertal mice. n=6 TEBs imaged in 4-5 mice per group ($p = 0.0875$, Welch's t test). **(G)** Representative confocal image of mutant GFP+ cells (in green) organized in a ring-like arrangement within a TEB 2 weeks after tamoxifen induction. Close up of Fig. 2N (blue box). Scale bar: 20μm. **(H)** Representative confocal images of optical sections showing cellular arrangements during the initiation and growth of aberrant buds in the SMACre/β-cat mammary epithelium. Top inset panels show DAPI staining (early displaced nuclei outlined in red in the top left inset). GFP fluorescence (left) and K5 immunostaining (right) are displayed in the bottom insets. Scale bars: 20μm. Right panel is a close-up of the bottom image in Fig.4I **(I)** TUNEL (brown) staining in SMACre/β-cat mammary sections indicating that cells within hyperplastic lesions undergo cell death. Nuclei are counterstained with Methyl green. Scale bar: 20μm. **(J)** Representative section of mammary gland tissues from SMACre/β-cat mice showing abundant cleaved-caspase 3 (CC3, red) immunostaining in an advanced lesion. GFP marks mutant cells in

green, anti-p63 staining labels basal/epidermal cells in white and DNA is stained by DAPI in blue. Scale bar: 20 µm.

Supplementary Figure 4

A

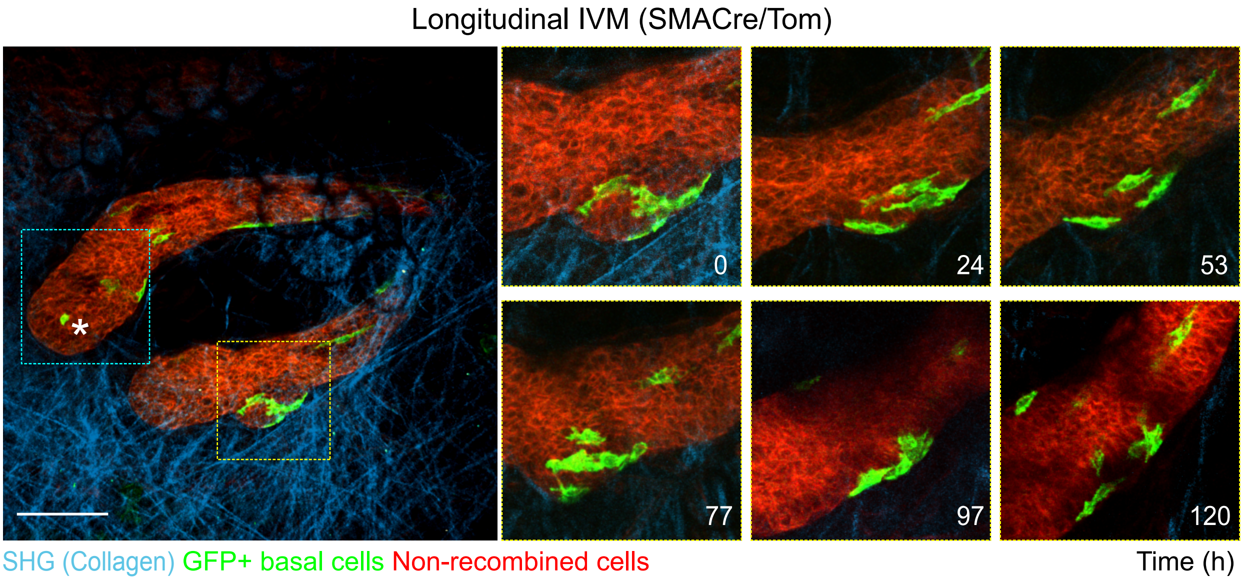

B

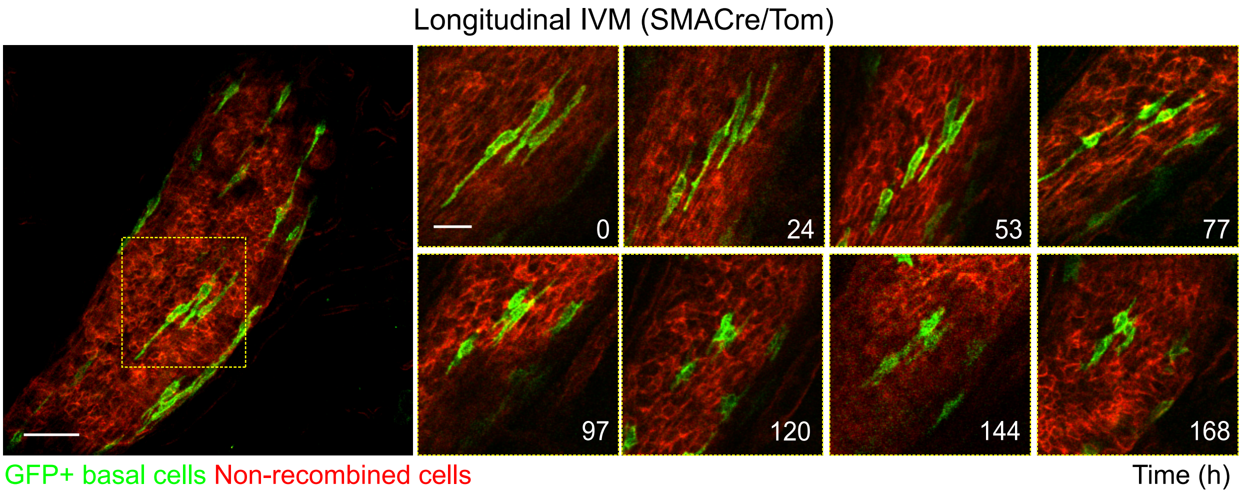

Fig. S4. Longitudinal IVM of wild-type β-cat basal cell behavior during pubertal ductal development. Related to Fig. 2.

(A) IVM images of a mammary terminal end bud (TEB) structure in a pubertal SMACre/Tom mouse showing recombined GFP+ (green) mammary basal epithelial cell rearrangements over time (120 h). The asterisk denotes cap-in-body cell shown in Fig.S4C. Red: non-recombined membrane tdTomato-expressing mammary epithelial cells; cyan: collagen (SHG). Area marked by blue box is included in Fig. S4C (top row). **(B)** IVM images of ductal basal GFP+ (green) cells in the mammary gland of a pubertal SMACre/Tom mouse over time (168 h). Red: non-recombined membrane tdTomato-expressing mammary epithelial cells. Scale bars: 100μm in A, 50μm in B (25μm in inset).

Supplementary Figure 5

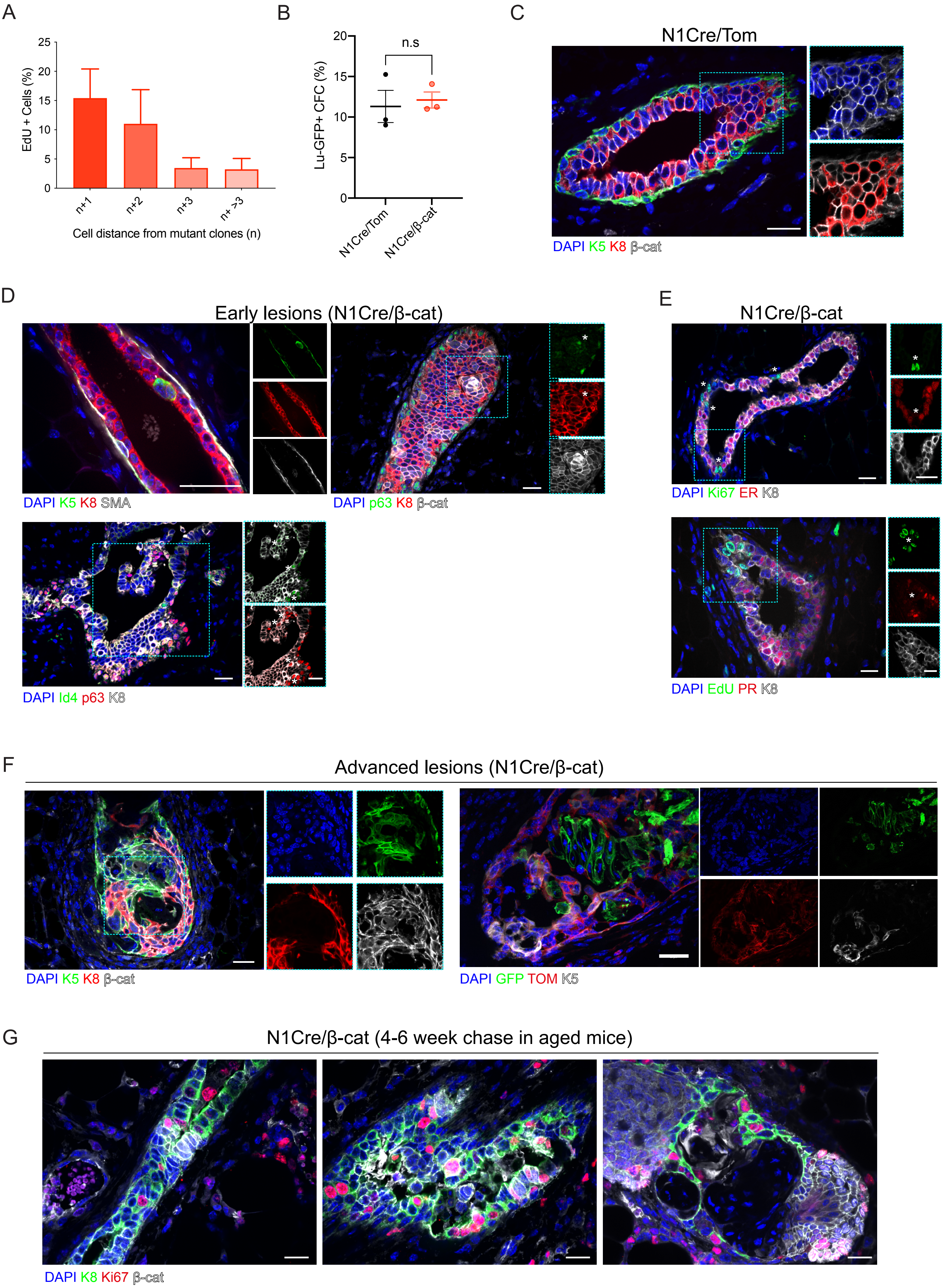

Fig. S5. β-catenin stabilization in mammary luminal cells leads to the development of lesions with aberrant lineage marker expression and squamous metaplasia. Related to Fig. 3.

(A) Positional analysis of EdU incorporation in N1Cre/β-cat mammary tissues. The graph shows the percentage of EdU+ cells observed at increasing cell distances from lesions displaying nuclear β-cat staining (as shown in Fig. 3C). Means $\pm$ SEM from 10 lesions in n=3 different animals. **(B)** Quantification of colony forming assay showing no differences in the colony forming capacity of GFP+ luminal cells isolated by flow cytometry from the mammary glands of N1Cre/Tom and N1Cre/β-cat mice. n.s: not significant. **(C)** Representative sections of mammary ducts in N1Cre/Tom mice showing β-catenin expression (white) restricted to the cell membrane. **(D)** Representative sections of N1Cre/β-cat mammary ducts showing acquisition of K5, p63 and Id4 basal marker expression (but not SMA) in hyperplastic lesions. **(E)** Representative sections of N1Cre/β-cat mammary ducts showing segregation of ER (red in upper panels) and PR (red in bottom panels) expression and proliferating (Ki67+ or EdU+) luminal cells (in green). anti-K8 immunofluorescence labels luminal cells in white and DNA is stained by DAPI in blue. Scale bars in C-E: 20μm. **(F)** Representative images of advanced lesions in mammary tissues of N1Cre/β-cat mice showing the formation of dysplastic, squamous-like structures with polymorphic nuclei, 3 (left panels) and 6 (right panels) weeks after tamoxifen induction. Scale bar: 20μm (left), 25μm (right). **(G)** Representative sections of N1Cre/β-cat mice induced at the age of 10 months and analyzed after 4 (left) or 6 (center, right) weeks of chase. Different lesion stages expressing nuclear β-catenin (in white) are shown: from a small ductal luminal lesion (left) to luminal hyperplasia (center) and squamous metaplastic K8-negative lesion (right). anti-K8 (in green) labels luminal cells and anti-Ki67 (in red) marks proliferative cells. Scale bars: 20μm

Supplementary Figure 6

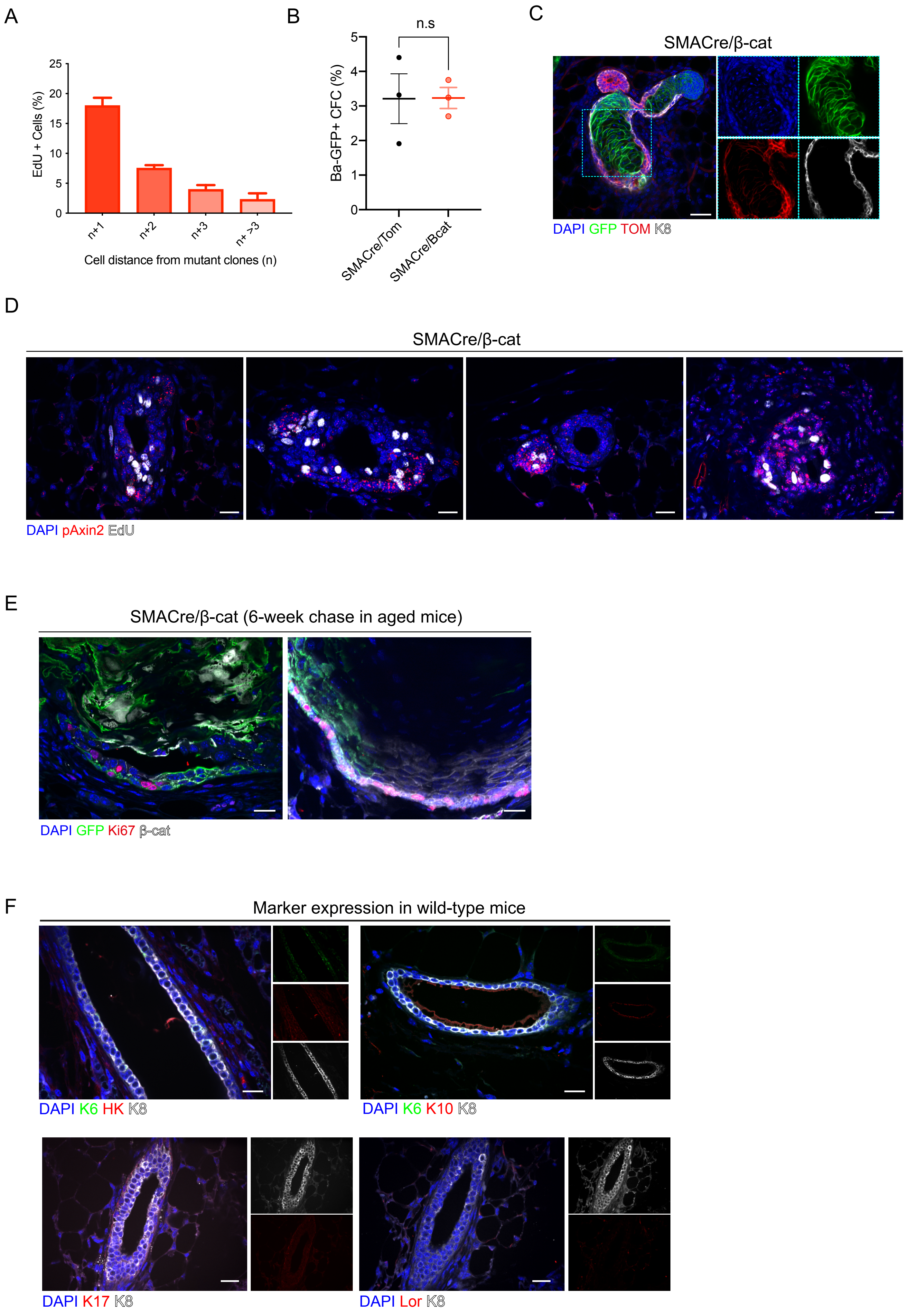

Fig. S6. β-catenin stabilization in mammary basal cells induces the development of rosette-like lesions and squamous metaplasia. Related to Figs. 4 and 5.

(A) Positional analysis of EdU incorporation in SMACre/β-cat mammary tissues. The graph shows the percentage of Edu+ cells observed at increasing cell distances from lesions displaying nuclear β-catenin staining (as shown in Fig. 4D). Means $\pm$ SEM from n=12 lesions in 4 different mice. **(B)** Quantification of colony forming assay showing no statistically significant differences in the colony forming capacity of GFP+ basal cells isolated by flow cytometry from the mammary glands of SMACre/Tom and SMACre/β-cat mice. n.s: not significant. **(C)** Representative image of an advanced lesion in a SMACre/β-cat mammary duct 20 days after TAM induction showing formation of squamous rosette-like structures with polymorphic aberrant nuclei. Scale bar: 50 µm. **(D)** Representative sections of SMACre/β-cat mammary tissues analyzed by smRNA FISH for Axin2 (red) and EdU incorporation (white). Related to Fig.4L. Scale bar: 20 µm **(E)** Representative sections of SMACre/β-cat mice induced at the age of 12 months and analyzed after 6 weeks of chase. A pilomatricoma-like tumor/cyst presenting nuclear β-catenin (in white) and high levels of Ki67+ cells (in red) is shown. GFP fluorescence denotes mutant cells. Scale bar: 20 µm. **(F)** Expression of differentiation markers of adult hair follicle and interfollicular epidermis in wild-type mammary tissues. Representative sections of mammary ducts from wild-type mice showing no detectable expression of hair keratin (HK), K10 and Loricrin (in red). Low levels of K17 (in red) were detectable in basal ductal cells. Luminal cells are marked in white by K8 immunofluorescence and DNA is stained in blue with DAPI. Scale bar: 20 µm.
